# Optimization of pretreatment and enzymatic hydrolysis using commercial and isolated bacterial enzyme cocktail for bioethanol production from corn husk through yeast co-culture batch fermentation

**DOI:** 10.1186/s40643-026-01062-z

**Published:** 2026-05-13

**Authors:** Barsha Samantaray, Rashmi Ranjan Mishra, Sonali Mohapatra, Sakti Rath, Bikash Chandra Behera, Hrudayanath Thatoi

**Affiliations:** 1 Department of Biotechnology, Maharaja Sriram Chandra Bhanjadeo University, Baripada, Odisha 757003 India; 2 Department of Biotechnology, MITS School of Biotechnology, Bhubaneswar, Odisha 751024 India; 3 Department of Biological Systems Engineering, Enzyme Research Institute, University of Wisconsin, Madison, USA; 4 Departmen of Life Sciences, Rama Devi Women’s University, Bhubaneswar, Odisha 751022 India; 5 School of Biological Sciences, NISER, Bhubaneswar, Odisha 752050 India; 6 Centre for Industrial Biotechnology Research, Siksha‘O’Anusandhan Deemed to Be University, Bhubaneswar, 751003 India

**Keywords:** Corn husk biomass, Ultrasonication pretreatment, Enzymatic hydrolysis, Enzyme cocktail, Fermentation, Bioethanol

## Abstract

This study assesses the impact of physicochemical pretreatment, enzymatic hydrolysis, and co-culture fermentation strategies on bioethanol production from corn husk biomass (CHB). Under optimal alkali pretreatment conditions (1.75% alkali, 4.0 g substrate concentration, 120 °C, 10 h), 35% lignin removal was achieved, with 48% cellulose and 39% hemicellulose recovery. In contrast, acid pretreatment resulted in 30% lignin removal, 45% cellulose recovery, and 34% hemicellulose recovery, showing lower efficiency than alkali pretreatment. During ultrasonication alkali pretreatment enhanced cellulose and hemicellulose exposure up to 51 and 46% and delignification up to 49%. Enzymatic hydrolysis of pretreated corn husk biomass was performed using commercial enzymes [Celluclast 1.5 L (700 EGU or 854 U mL^−1^) and Viscozyme (13.4 FBG/mL)] and isolated bacterial enzymes, including cellulase from *Bacillus licheniformis* (9.3 ± 0.3 U mL^−1^) and xylanase from *Enterobacter asburiae* PQ396173 (7.0 ± 0.4 U mL^−1^). The developed enzyme cocktail in ratio 3:2:3:1 (v/v; U mL^−1^) (Celluclast: Viscozyme: native cellulase: native xylanse) using a cocktail of native and commercial enzymes, yielded total reducing sugar of 740 mg g^−1^ glucose and 54.6 mg g^−1^ xylose. Fermentation of hydrolysate prepared with commercial enzymes using monoculture of *Saccharomyces cerevisiae* and *Pichia pastoris* yielded 17.6 g L^−1^ and 12.2 g L^−1^ bioethanol separately. Co-cultured yeasts produced 26.8 g L^−1^ ethanol at 96 h of incubation, exceeding monoculture yields. The fermentation with integration of commercial and isolated bacterial enzyme cocktails yielded the highest bioethanol output of 37.3 g L^−1^ at 96 h incubation, indicating that enzymatic saccharification with a combination of commercial and native enzyme cocktails results in maximum bioethanol production.

**Graphical abstract:**

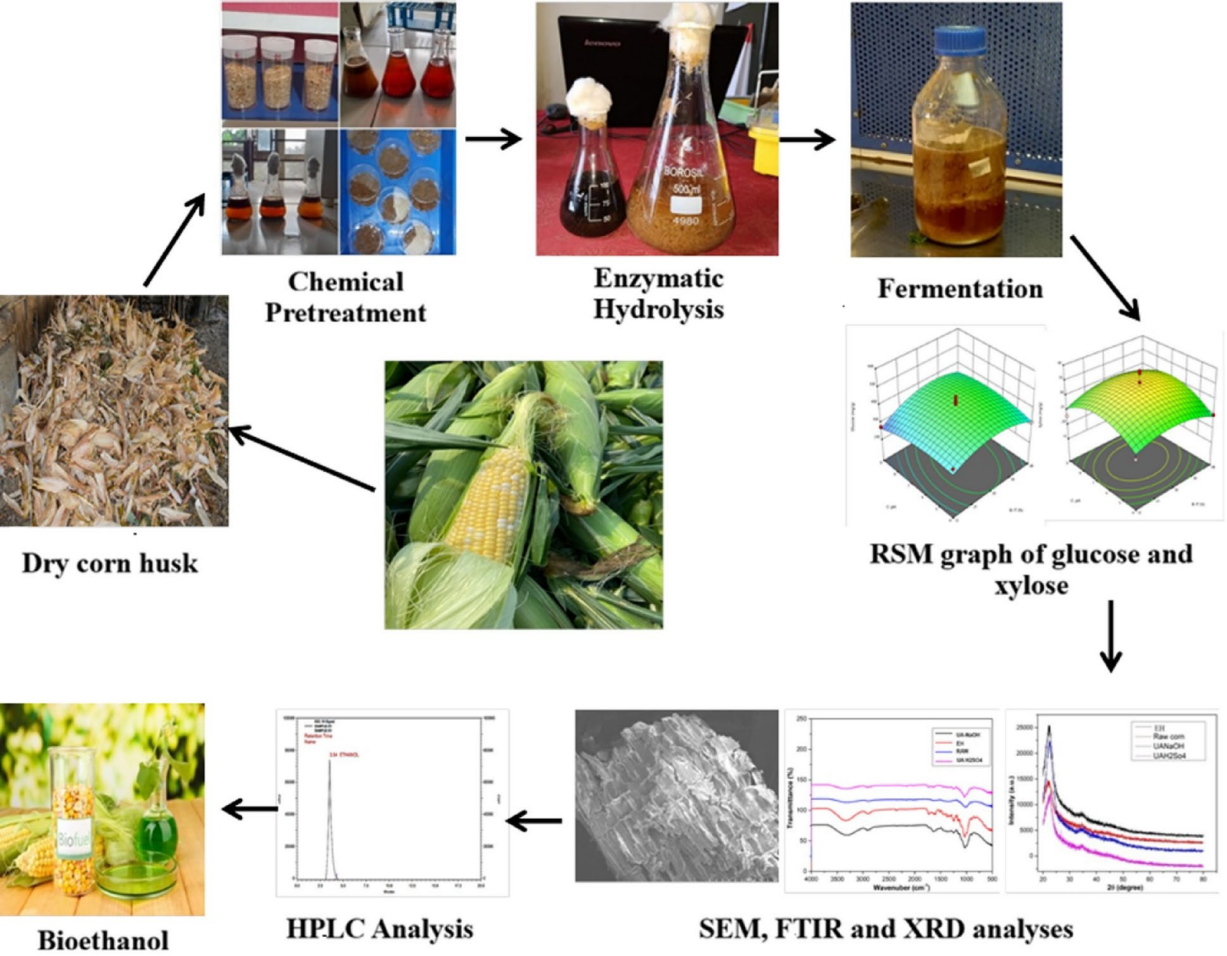

**Supplementary Information:**

The online version contains supplementary material available at 10.1186/s40643-026-01062-z.

## Introduction

Sustainable energy resource utilization has become crucial because excessive fossil fuel use significantly harms the environment and depletes fossil fuel supplies (Anand et al. 2023). Researchers now use lignocellulosic biomass as the most common and carbon–neutral feedstock for biofuel production (Nahak et al. 2022; Zhang et al. 2023). Farmers produce corn husk in large quantities as an agricultural residue, which can be converted into biofuels through various biochemical processes (Li et al. 2022). Bioethanol is the leading biofuel, accounting for around two-thirds of global biofuel production. This renewable energy source has gained significant attention worldwide due to its feasibility, eco-friendliness, and high-octane number, making it a viable alternative or blending with petrol (Sindhu et al. 2019; Punia and Kumar 2025).

Many countries generate abundant corn husk biomass (CHB), which provides enormous potential for bioenergy generation (Aghaei et al. 2022). In India, maize ranks as the third most important crop after wheat and rice, contributing about 9% to the country's total food grain production. Farmers use about 60–65% of India's maize for cattle and poultry feed. Food processing industries consume 10–15% of products such as cornflakes, dextrose, starch, popcorn, corn oil, and corn syrup. Consumers use the remaining 20–25% for dietary purposes (Murdia et al. 2016). As maize cultivation expands substantially in India and globally, farmers generate large amounts of agricultural debris, including corn husks, corn stover, and corn cobs. Researchers recognize corn cobs and husks as significant lignocellulosic wastes for bioethanol production (Sokan-Adeaga et al. 2024). These materials consist of high hemicellulose content, and they contain less lignin and ash compared to other types of biomass (Gandam et al. 2022). Corn husk biomass is the largest source of fermentable sugars and can be converted into ethanol due to its high cellulose content, which consists of approximately 45.7% cellulose, 35.8% hemicellulose, and 4.3% lignin (Ibrahim et al. 2019).

During the conversion process of bioethanol, the lignin in the biomass raises significant hurdles for the enzymatic hydrolysis into fermentable sugars, as it serves as a physical barrier that obstructs the ability of cellulase to interact with cellulose. This resistance makes the hydrolysis process rate-limiting (Li et al. 2019). Thus, corn biomass energy has grown to be a significant problem that requires sustainable solutions (Xie et al. 2022). For this reason, various approaches have been undertaken for the delignification of corn biomass waste that require less energy, are cost-effective, renewable, and can be sustainably utilized to produce bioethanol (Elsagan et al. 2023).

Various pretreatment techniques have been employed in bioethanol production to remove lignin from corn husks. These techniques include chemical treatments, ionic liquids, organosolv processes, and Ultrasono assisted pretreatment (Wang et al. 2024). Standard chemical pretreatment techniques using acids or alkalis are preferred for lignin removal from corn husk due to their lower cost and higher efficiency. It has been reported that lignin can be dissolved from lignocellulosic biomass through size reduction followed by acid hydrolysis (using HCl, H_2_SO_4_, etc.) or with alkalis like NH_3_ or NaOH. Ultrasound is a sound wave that may agitate and create cavitation in liquids, disrupting the surface structure of organic cellulose biomass. The cellulose and hemicellulose are separated from the lignocellulosic biomass by hydrolysis after pretreatment to get the fermentable sugars (Samantaray et al. 2024a).

After effective pretretament, enzymatic hydrolysis significantly enhances cellulose conversion to simple sugars. Cellulase enzymes convert cellulose to glucose under various conditions, subject to the elimination of lignin. Thus, pretreatment should be designed to balance the high digestibility of biopolymers with low inhibitor production using hydrolytic agents, such as lignocellulolytic enzymes (ligninases, cellulases, and xylanases). It is advised to use microbial enzymes for hydrolysis as a pretreatment since it lowers manufacturing costs, increases conversion rate, and causes the least amount of environmental harm, making it eco-friendly (Rana et al. 2019; Sharif et al. 2023). Cellulose, a linear homo-polysaccharide, is hard to break down due to hydrogen bonds within and between its chains. Its complex interactions with lignin and hemicelluloses further hinder hydrolysis. While dealing with hemicelluloses is relatively manageable, breaking down cellulose, whether delignified or still linked with lignin, poses a significant industrial challenge (Devi et al. 2022). Breaking down hemicelluloses enzymatically or removing them during pretreatment isn't a big issue. The real challenge at an industrial scale is hydrolyzing cellulose, whether it's been de-lignified or still has lignin attached. The conversion of biomass to biofuels involves three primary stages: pretreatment, enzymatic hydrolysis, and fermentation of sugars. Notably, pretreatment and enzymatic hydrolysis, including enzyme costs, are identified as the major cost contributors in this process (Adsul and Gokhale 2012; Jin et al. 2016). The complete conversion of lignocellulosic biomass to fermentable sugars necessitates the involvement of multiple enzymes, highlighting the complexity of the process (Puri et al. 2013). Effective hydrolysis of pretreated biomass relies heavily on the quality of enzyme preparations. Notably, microorganisms that produce the entire range of enzymes necessary for efficient lignocellulosic biomass hydrolysis are relatively rare, underscoring a significant challenge in the process. The development of efficient and cost-effective enzyme cocktails for cellulose hydrolysis remains a pivotal research focus in biomass conversion, aiming to enhance process economics (Adsul et al. 2020).

The most common method in bioethanol production is the simultaneous saccharification and fermentation (SSF). This technique is similar to the separate hydrolysis and fermentation methods, but SSF integrates these two processes by conducting fermentation and hydrolysis simultaneously (Valles et al. 2020). SSF was employed to decrease processing times and increase the output rate. It can convert sugars more quickly, reducing the need for enzymes, increasing the product yield, and requiring inadequate sterilization (Karimi et al. 2021). Many yeast species are known to generate extracellular enzymes that lead to alcohol production, including *S. cerevisiae, Pichia stipitis,* and *Candida shehatae* (Kathiresan et al. 2011). Enzyme activity is reduced when fermentation is conducted with a single microbe. However, the co-culture consortium using different microorganisms could improve their interactions and reduce the chosen strain's enzyme synthesis restrictions (Kundu et al. 2019). To enhance the metabolic process, two or more microorganisms must be co-cultured, which is a complex process that requires controlling and promoting contact between the chosen organisms to complete two metabolic processes.

This limitation significantly hinders the industrial application of *S. cerevisiae*, where efficient ethanol production from lignocellulosic biomass is a key objective. Furthermore, *S. cerevisiae* is incapable of fermenting pentose sugars, notably xylose, present in lignocellulosic substrates, due to the absence of the requisite metabolic mechanism, specifically the oxidoreduction pathway, necessary to convert xylose to xylulose for entry into the pentose phosphate pathway (PPP) (Song et al. 2020). Co-culturing of non-conventional pentose-fermenting yeast species with *S. cerevisiae* in biofuel fermentation processes offers a promising approach to augment bioethanol yields from xylose-containing biomass substrates (Wu et al. 2023). In this context, co-culturing of *S. cerevisiae* with *Pichia* sp. gives better ethanol output.

With these insights, the present study aimed for the optimization of chemical pretreatment by using the RSM technique. Further, enhancement of delignification, the ultrasono assisted alkali pretreatment technique was employed and showed better results. In order to address these challenges of the saccharification process, in this article, we discussed the necessity of the formulation of an enzyme cocktail using crude and commercial cellulase and xylanase enzymes for higher saccharification and to enhance the total reducing sugar yield. Additionally, simultaneous saccharification and co-culture fermentation (SSCF) using two yeast strains, i.e., *S. cerevisiae* (MTCC-170) and *P. pastoris* (MTCC-34), was used for bioethanol production.

This study differs from existing corn husk based bioethanol research by integrating native microbial enzymes with commercial enzyme formulations to enhance biomass hydrolysis and ethanol production. Unlike earlier studies that relied solely on commercial enzymes, our approach harnesses the synergistic activity of indigenous enzymes, resulting in improved hydrolysis efficiency and higher bioethanol yield. Moreover, this strategy reduces enzyme dependency and associated costs while improving process sustainability, highlighting the novelty and practical significance of our work in lignocellulosic bioethanol production.

## Materials and methods

### Preparation of raw corn husk biomass

Corn husks were collected from a local agricultural field in the Khordha district of Odisha, India. To remove any undesirable dust particles, the biomass was rinsed twice with deionized water. After that, it was oven-dried at 100 °C for 4 h. After drying, the biomass was ground using a ball mill and sieved to make a particle size of 2–3 mm (Ding et al. 2023). The biomass was securely preserved in the polyethylene bags for further studies.

### Optimization of pretreatment techniques

For optimizing the sulfuric acid (H_2_SO_4_) and sodium hydroxide (NaOH) pretreatment process, Response Surface Methodology (RSM) (Design Expert software version 13.0) with a three-level, four-factor Box-Behnken design was used. A total of thirty-one runs were performed, adjusting four parameters: substrate concentration (g L^−1^), incubation time (min/h), temperature (°C), and acid/alkali concentration (%, v/v). The experimental data were fitted using a quantitative model, and ANOVA was performed to evaluate the coefficients. The model’s validity was assessed by comparing experimental and predicted values. For acid pretreatment, the factors included incubation time (40, 50, and 60 min), temperature (60, 90, and 160 °C), substrate concentration (SC) (2, 3, and 4 g), and acid concentration (AC) (0.5, 1.25, and 3.0%). For alkali pretreatment, factors were incubation time (4, 7, and 10 h), temperature (50, 85, and 120 °C), substrate concentration (SC) (2, 3, and 4 g), and alkali concentration (ALKC) (0.5, 1.75, and 3%). The chosen ranges of each factor for pretreatment were considered following the methodology of Mohapatra et al. (2020) and Mishra et al. (2020). Finally, the concentrations of lignocellulosic components were estimated using the standard protocol. The substrate loading was 4 g/100 mL throughout all trials, with experiments conducted in separate 150 mL flasks at 120 °C. After thorough washing and filtration through muslin cloth, the biomass was oven-dried. The amounts of cellulose, hemicellulose, and lignin in the solid fractions were measured, following the method of Mohapatra et al. (2020).

### Ultrasonic pretreatment

Sonication was conducted using an ultrasonicator (Bandelin Sonoplus HD 2070, Germany) at 400 W and 24 kHz. The CHB biomass was dried in an oven overnight at 105 °C to remove the water content. In a flask, 100 mL of a 3% (w/v) alkali solution was heated to 50 °C, and 100 g of CHB was added at a 1:20 (w/w) ratio. Ultrasonic radiation was applied through a titanium probe submerged 10 mm into the mixture for 60 min, followed by rapid cooling with tap water. The mixture was filtered to separate the liquid from the solid residue, which was then rinsed with distilled water until neutral pH. Finally, the solid residues were dried until the desired weight was achieved.

### Microorganisms

A cellulose-producing bacterial strain was isolated from cow dung locally collected from the cowshed of Bhubaneswar, Odisha. Morphological, biochemical, and molecular characterization of the bacterial strain was done, and it was identified as *Bacillus licheniformis*. The xylanase-producing bacterial strain *Enterobacter asburiae* PQ396173 was collected from the Department of Biotechnology, MSCBU, Baripada, Odisha, and further evaluated for xylanase assay.

### Assay for cellulase and xylanase activity

The bacterial strain's cellulase activity was determined using the Dinitrosalicylic acid test (DNSA), which was adapted from Sharma's (2019) methodology. For this, 0.5 ml of cellulase was diluted with an equal volume of citrate buffer (0.05 M, pH 4.8). 0.5 mL of the substrate (1% CMC) solution was added to 1 mL of pre-diluted cellulase and was incubated for 30 min at 40 °C. Finally, 3 mL of DNS reagent was added to each test tube and mixed thoroughly. Then the test tubes were kept in a water bath at 100 °C for 15 min. After incubation, the test tubes were allowed to cool down at room temperature, and the absorbance was measured at 540 nm using a UV–visible spectrophotometer. The xylanase activity of the bacterial strain *Enterobacter asburiae* was performed following the method of Irfan et al. (2016). The reaction mixture, which contained 0.5 mL of xylanase and 0.5 mL of a 1% xylan (beech wood) solution made in citrate buffer (0.05 M, pH 4.8), was incubated at 35 °C for 30 min. Following the incubation period, 5 mL of Bial's reagent was added and left in a boiling water bath for 10 min to stop the reaction. Following cooling, spectrophotometric measurements of the released reducing sugars were made at 620 nm.

Enzymatic hydrolysis experiments were carried out to evaluate the saccharification efficiency of pretreated lignocellulosic biomass. The hydrolysis reactions were performed using crude or partially purified cellulase and xylanase enzymes. The enzyme activities were determined prior to application and expressed in units per millilitre (U/mL), where one unit corresponds to the amount of enzyme required to release 1 µmol of reducing sugar per minute under standard assay conditions. Pretreated dry biomass (4 g) was incubated with a fixed volume of enzyme solution (mL) with appropriate proportions under various optimized conditions of pH, temperature, and incubation time. To ensure reproducibility and enable comparison across treatments, enzyme loading was normalized on a dry biomass basis and expressed as U/g biomass using the following equation:

$$ \begin{aligned} & {\text{Enzyme loading }}\left( {{\mathrm{U}}/{\text{g biomass}}} \right) \\ & \quad = \frac{\begin{array}{l}{\text{Enzyme activity }}\left( {{\mathrm{U}}/{\mathrm{mL}}} \right) \\ \quad \times {\text{Volume added }}\left( {{\mathrm{mL}}} \right) \\ \end{array} }{{{\text{Dry biomass }}\left( {\mathrm{g}} \right)}} \\ \end{aligned} $$

All the hydrolysis experiments were optimized using RSM technique, and the released sugars (glucose and xylose) were quantified using standard analytical methods. The results were expressed as mg/g biomass.

### Optimization of the enzymatic hydrolysis process by RSM

The enzymatic hydrolysis process was carried out using both commercial enzymes (Celluclast 1.5 L (700 EGU), Viscozyme (13.4 FBG) and isolated bacterial cellulase and xylanase enzymes from *Bacillus licheniformis* (U mL^−1^) and *Enterobacter asburiae* PQ396173 (U mL^−1^) were used. Throughout the experiments, a substrate loading of 4 g/100 mL in citrate buffer was maintained. The process was optimized using RSM with various factors such as pH (4.8, 5.5, 6.2), enzyme ratios (v/v; U mL^−1^) (cellulase and xylanase = 1:1, 1:2, 1:3, 2:1, 2:2, 2:3, 3:1, 3:2 and 3:3), incubation times (24, 36, and 48 h), and temperatures (40, 45, and 50 °C). Finally, the concentration of reducing sugar was estimated using the method described by Miller (1959). A total of 27 experimental runs were conducted, and the samples were analyzed using ANOVA. The model's adequacy was confirmed through a prediction test comparing predicted and experimental values of total reducing sugar concentration (TRS).

### Analytical techniques

The cellulose content of the hydrolyzed biomass was measured using the standard method described by Updegraff (1969). Hemicellulose content in the sample was quantified using the nitric acid reagent technique (Roe and Rice 1948). The acid-soluble and insoluble lignin was estimated following the standard method of Sluiter et al. (2008). Bioethanol concentration was determined with a dichromate assay (Tupe et al. 2018). Ethanol standards were prepared using 10% ethyl alcohol, adding 1 mL of the test sample and 2 mL of chromic acid. The solution was incubated at 50 °C for 20 min, and the absorbance was measured at 600 nm.

### Characterization of the raw and treated biomass

The structural characteristics of both native and chemically pretreated corn husks were analyzed using a Scanning Electron Microscope (SEM) (Hitachi, Model: S-3400, USA). Fourier Transform Infrared (FTIR) spectroscopy was performed for structural vibrational analysis with 44 scans at a resolution of 2 cm^−1^. X-ray powder diffraction (XRD) was conducted using a diffractometer (X'PERT PRO, PAN analytical, Netherlands) with a Cu Kα radiation source.

### Inoculation development for fermentation

For fermentation, *Saccharomyces cerevisiae* (MTCC-170) and *Pichia pastoris* (MTCC No. 34) were purchased from Microbial Type Culture Collection and Gene Bank (MTCC), Chandigarh, India. They were maintained at the Biotechnology Department of MITS Bhubaneswar. They were cultured in the Yeast extract Peptone Glycerol (YPG) medium (Himedia) to activate the yeast strains. The strains were inoculated separately in a 250 mL conical flask with 100 mL of YPG medium (pH 5.6). The growth pattern of both yeast strains was studied for up to 24 h until the OD value reached 0.8–1.0 at 600 nm. At the exponential growth phase of the culture, the yeast cells were harvested by centrifugation at 8000 rpm. Then, the fermentation experiment was carried out using pretreated and hydrolyzed CHB as substrate. Every 12-h interval, aliquots were taken, and the concentrations of the reducing sugars (glucose and xylose) were measured by the dinitrosalicylic acid method (DNS), and the bioethanol content was estimated by dichromate assay, using the colorimetric quantification based on the formation of green colored chromate ions resulting from the treatment of ethanol. The absorbance maxima for the ethanol were found to be 578 nm (Tupe et al. 2018).

#### Simultaneous saccharification and co-culture fermentation

After enzymatic hydrolysis, the initial sugar content in the hydrolysate was approximately 46 g L^−1^, was taken in 500 mL screw cap reagent bottles and agitated at 100 rpm. Both the yeast strains *S. cerevisiae* and *P. pastoris* were inoculated simultaneously during their exponential phase of growth, such as 48 h incubation time, 6.5 pH, and 32 °C, and were inoculated into the fermentation medium. YPG medium (1 g of yeast extract, 2 g of peptone, and 2 g of dextrose) supplemented with CHB and without yeast inoculums was treated as a control. Several experimental parameters, including temperature (28, 30, 32, 34, 36, 38, and 40 °C), pH (4.5, 5, 5.5, 6.5, and 7), incubation period (12, 24, 36, 48, 60, 72, 84, and 96 h), and inoculums volume ratios (1:1, 2:1, and 3:1) were used to optimize the fermentation. Commercial cellulase and xylanase enzymes in different ratios (1:1, 2:1, and 3:1) were also used. The pH is typically controlled and maintained within an optimal range of 4.5–7.0 over the 96 h of incubation period by the addition of buffers and periodic adjustments with 1N HCL and 1N NaOH.

#### Detection and quantification of ethanol using HPLC

After fermentation, the distilled samples were collected and analyzed using HPLC (Kuila and Banerjee 2014) (Agilent Technologies 1260 Infinity Series) with a Quaternary pump 1260 Quat. Pump VL, 1260 ALS Autosampler, 1260 TCC column oven, a Zorbax Carbohydrate Analysis Column (4.6 mm ID × 250 mm, i.e., 5 μm particle size), and a 1260 Refractive Index Detector. Using Open Lab CDS EZ Chrom system controllers, each component of the chromatographic system was managed. Data validation was carried out using Open Lab CDS Data Analysis version A.01.01 software.

## Results and discussion

### Characterization of corn husk biomass

The raw CHB was primarily characterized by its total cellulose, hemicellulose, and lignin (C–H–L) content. The experiment revealed that the raw corn husk biomass contains 46 ± 2.1% cellulose, 32 ± 1.5% hemicellulose, and 13 ± 3.1% lignin. Before converting the biomass into ethanol, it is essential to determine the composition of the lignocellulosic substrate. In a similar type of experiment, Pointner et al. (2014) reported that the C–H–L contents of corn cob dry matters were 39 ± 2.5%, 44 ± 5.2%, and 12 ± 2.3%, respectively. Similarly, in another study made by Behl et al. (2023), corn stalks were found to contain 35% cellulose, 28% hemicellulose, and 22% lignin.

### Optimization of chemical pretreatment

**Fig. 1:**
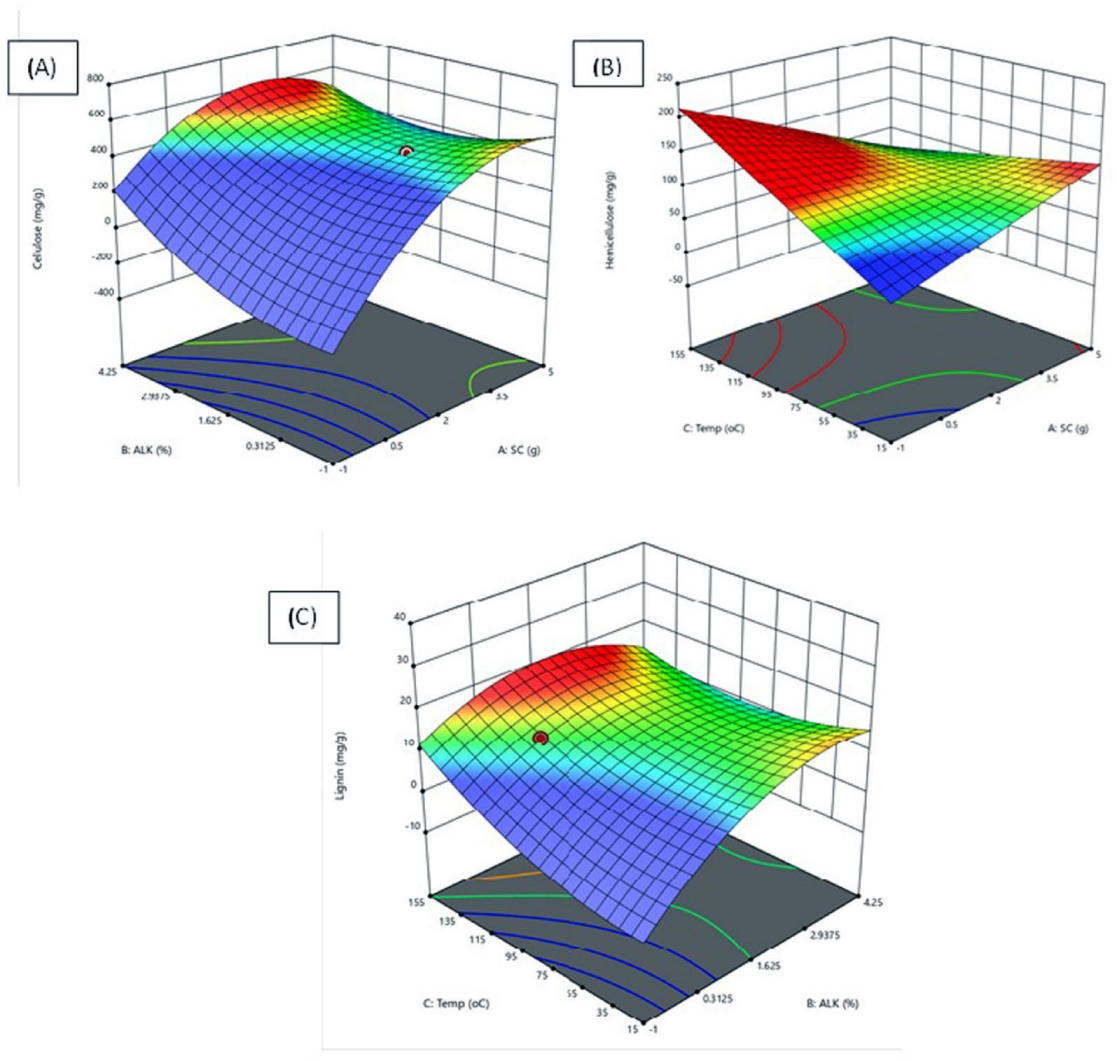
RSM graph showing effect of process parameters on the recovery of **A** Cellulose, **B** Hemicellulose and **C** lignin for Alkaline (NaOH) pretreatment

**Fig. 2:**
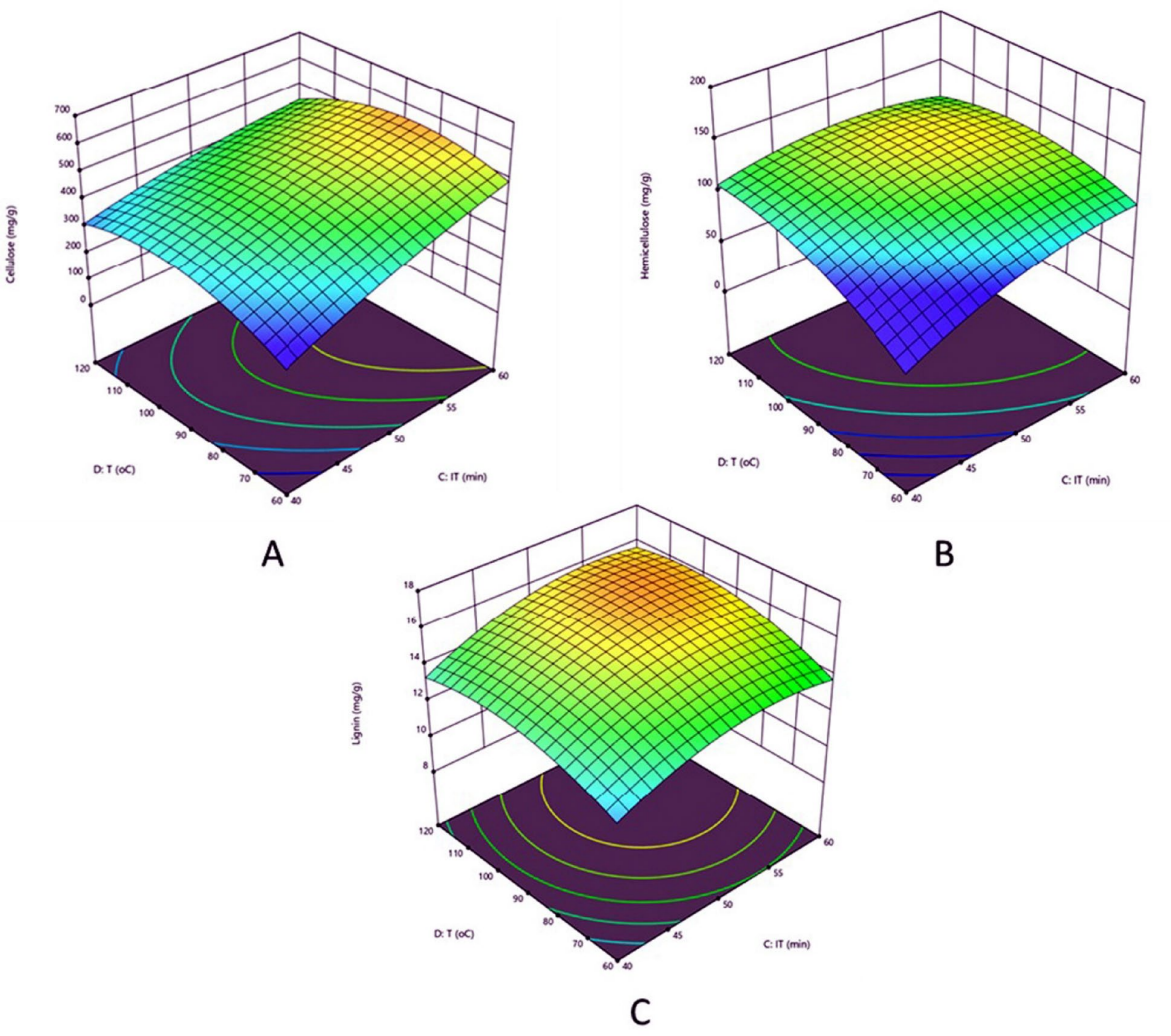
RSM graph showing effect of process parameters on the recovery of **A** Cellulose, **B** Hemicellulose and **C** lignin for Acid (H_2_SO_4_) pretreatment

For the breakdown of lignocellulosic complex and exposure of cellulose, both physicochemical pretreatment methods were adopted. This usually increases the amount of cellulose available for enzymatic hydrolysis. RSM-based optimization of acid and alkaline pretreatment was applied to the CHB. A quantitative model was utilized to fit the experimental data. Table 1 represents the process variables and their corresponding levels used in the experimental design. For alkali pretreatment using sodium hydroxide (NaOH), the following conditions were used to maximize the exposure of cellulose, hemicellulose, and lignin: a substrate loading of 4 g, a temperature of 120 °C, an incubation time of 10 h, and a NaOH concentration of 1.75% were used. Under these optimal conditions, the maximum yields of 687 mg g^−1^ cellulose, 177 mg g^−1^ hemicellulose, and 18 mg g^−1^ lignin were achieved (Table 2 and Fig. 1). Similarly, sulphuric acid (H_2_SO_4_) pretreatment was performed to expose C-H–L content. The highest cellulose obtained was 609 mg g^−1^ of CHB under optimum conditions, including an incubation time of 60 min, a temperature of 150 °C, SC of 2 g, and an acid concentration of 2% (v/v) (Table 3 and Fig. 2). The results indicated that alkali pretreatment produced a greater cellulose content compared to the diluted acid pretreatment under optimal conditions. In a similar type of study performed by Selvakumar et al. (2022), a maximum cellulose recovery of 93.5 ± 1.3% was reported, along with an 85.6 ± 1.8% removal of hemicellulose and 81.4 ± 2.3% removal of lignin in acid-pretreated corn cobs. ANOVA was conducted to determine the coefficients of the variables. The model's validation involved comparing the predicted values with the experimental values for both acid and alkali pretreatment of the CHB. After pretreatment, the results of ANOVA for the maximum exposure of cellulose, hemicellulose and delignification are presented in Tables S1, S2 and S3.

**Table 1:** Coded level of variables selected using Box-Behnken design

Coded factor	Factors name	Coded levels		
		− 1	0	+ 1
		Actual levels (Acid pre-treatment)		
A	Substrate Conc. (g)	2	3	5
B	Acid Conc. (%)	0.5	1.25	2
C	Incubation time (min)	40	50	60
D	Temperature (°C)	60	90	120

Coded factor	Factors name	Actual levels (Alkali pre-treatment)		
A	Substrate Conc. (g)	2	3	4
B	Alkali Conc. (%)	0.5	1.75	3
C	Temperature (°C)	4	7	10
D	Incubation time (h)	50	85	120

**Table 2:** Alkaline pretreatment of corn husk showing different concentration of cellulose, hemicellulose and lignin

Sl No	Substrate conc (g)	Alkali conc (%)	Incubation time (h)	Temperature (°C)	Cellulose (mg g^−1^)	Hemicellulose (mg g^−1^)	Lignin (mg g^−1^)
1	3	1.75	4	85	395	109	15
2	2	3.0	7	85	502	106	13
3	4	0.5	10	120	457	78	12
4	2	0.5	10	50	371	120	15
5	2	3.0	4	50	439	74	12
6	3	1.75	4	120	517	121	13
7	4	3.0	7	85	510	110	14
8	3	1.75	10	120	477	105	11
9	3	1.75	7	85	591	111	16
10	2	3.0	10	85	465	89	15
11	4	3.0	10	50	429	105	13
12	2	3.0	10	50	397	101	17
13	4	3.0	4	50	366	93	18
14	3	1.75	4	120	445	109	9
15	2	0.5	7	85	457	77	9
16	4	1.75	10	50	575	119	14
17	3	1.75	7	85	488	82	8
18	3	1.75	7	85	514	76	8
19	4	0.5	7	85	531	83	7
20	4	0.5	4	50	505	91	7
21	4	3.0	4	120	383	88	17
22	3	1.25	4	50	411	97	12
23	3	1.75	7	85	519	101	7
24	3	1.75	7	15	362	89	18
25	3	1.75	7	155	420	125	14
26	3	0.75	13	85	448	92	13
27	2	0.5	7	85	515	78	13
28	4	1.75	10	120	687	177	18
29	2	0.5	10	120	434	97	12
30	3	1.75	7	85	595	109	15
31	2	0.3	7	85	402	106	10

**Table 3:** Acid pretreatment of corn husk showing different concentration of cellulose, hemicellulose and lignin

Sl No	Substrate conc (g)	Acidic conc (%)	Incubation time (min)	Temperature (°C)	Cellulose (mg g^−1^)	Hemicellulose (mg g^−1^)	Lignin (mg g^−1^)
1	2	0.5	40	120	374	121	13
2	3	1.25	50	90	382	120	14
3	4	0.5	40	120	560	125	14
4	2	2.0	60	150	599	131	15
5	4	2.0	40	120	391	128	16
6	2	2.0	40	120	528	120	14
7	5	1.25	50	90	466	123	14
8	3	1.25	50	90	363	97	14
9	2	0.5	60	120	583	112	16
10	2	0.5	40	120	374	117	12
11	4	2.0	40	120	474	131	16
12	2	2.0	60	120	383	74	13
13	2	2.0	60	150	609	145	17
14	4	0.5	40	120	220	99	14
15	3	1.25	50	90	365	119	14
16	3	1.25	50	90	425	107	16
17	3	2.75	50	150	340	129	14
18	3	1.25	50	90	388	125	17
19	4	2.0	60	120	355	115	14
20	3	1.25	50	150	309	116	15
21	3	1.25	70	90	394	94	17
22	3	1.25	50	150	349	132	15
23	4	2.0	60	60	329	95	11
24	3	1.25	50	90	463	96	16
25	3	0.25	50	150	580	123	14
26	3	1.25	50	90	455	122	14
27	3	1.25	50	90	386	132	12
28	4	0.5	60	120	439	105	17
29	1	1.25	50	90	466	110	16
30	4	0.5	60	60	520	92	15
31	2	2.0	40	120	445	120	11

### Factors influencing the pretreatment process

This study evaluates the impact of various elements on the removal of lignin (mg g^−1^) and the increase in cellulose (mg g^−1^) and hemicellulose (mg g^−1^) during alkaline pretreatment. The maximum cellulose yield of 686.2 mg g^−1^ was achieved under the following conditions: 10 h of incubation time, 120 °C, 4 g of solid lignocellulosic biomass, and 1.75% alkali concentration (Fig. 1A). Among the four factors studied, solid biomass concentration (SC) and Alkali concentration had the most significant effect on cellulose exposure. The highest quantities of hemicelluloses, and lignin were observed at 177 mg g^−1^ and 18 mg g^−1^ under the same parametric conditions. In contrast, temperature (T) and SC were primarily responsible for the hemicellulose content (Fig. 1B), while T and alkali concentrations were the main factors influencing the delignification process (Fig. 1C). A study conducted by Mishra et al. (2020) revealed that factors such as alkaline concentration, temperature, incubation time, and substrate concentration significantly impact the delignification of banana peels during biobutanol production. In that study, the highest cellulose yield recorded was 530 mg g^−1^, achieved at 60 min, 40 °C, 6 g of SC, and 1.25% alkali concentration. Similar to our findings, the results indicated that SC and alkali concentration had the most significant impact on cellulose exposure compared to other parameters. Overall, it was observed that the concentration of SC and alkali significantly influenced the cellulose, hemicelluloses, and lignin (%) content after NaOH pretreatment. After alkali pretreatment, it was found that there was an increase in cellulose (38%), hemicellulose (41%), and lignin (14%) exposure as compared to the raw corn husk biomass. After alkali pretreatment, Mensah et al. (2021) observed that the highest cellulose content in corn stover was 81%, and the lowest was 67% in corn leaves. Similarly, the highest percentage of delignification was recorded at 85% in cornstalk, and the lowest at 53%. Further, the acid (H_2_SO_4_) pretreatment was applied to remove lignin and expose cellulose and hemicellulose from raw biomass. Various factors involved and affected during the delignification of biomass and release of cellulose and hemicellulose of acid pretreatment are shown in Fig. 2. Under optimal conditions, the factors include 60 min of incubation, 90 °C temp. With 2 g of SC and 2% (v/v) of AC, the maximum cellulose content was found to be 609 mg g^−1^ of pretreated biomass. It was found that temperature and IT are the two factors that substantially affect cellulose exposure (Fig. 2A). In the same parametric conditions, hemicellulose was recorded at 145 mg g^−1^, and delignification was found to be 17 mg g^−1^, respectively. Among the various factors studied, temperature and incubation time showed a significant impact on the release of hemicellulose as well as the delignification of CHB (Fig. 2B and C). It was interesting to discover that the factors mostly affected by maximal cellulose exposure and delignification were temperature and IT. Previous research showed that sulphuric acid pretreatment is a standard process for the removal of lignin and the release of cellulose and hemicellulose from raw biomass.

The ANOVA results were used to determine differences between more than two groups. The 3D models for C–H–L have shown a higher *F*-value (Fisher’s variance) with a significant *P*-value (probability). Analysis of the data presented in Table S4 via ANOVA revealed a statistically significant difference (*p* < 0.05) in cellulose percentage attributable to the primary constituent. Consideration would be given to four primary parameters: Substrate concentration, the NaOH/H_2_SO_4_ concentration, temperature, and incubation time, with the interplay of these elements having an effect on the percentage of cellulose content. Table S4 illustrates that the beneficial correlation between particle size and NaOH concentration with time had a significant impact on the concentration of cellulose. This interaction was quite significant. Consequently, the application of *P*-value and *F*-value, which are statistical terms used to assess model and variable significance, substantiates the importance of the components, indicating their considerable relevance. A *P*-value less than 0.05 indicate a 95% probability of significance at the level of each model. In this study, the model terms AC, AB, BC, A^2^, and C^2^ are statistically significant. Conversely, terms with values exceeding 0.1 are deemed insignificant. The interactions between AB, AC, and BC are significant because their *P*-values are less than 0.001, 0.006, and 0.007, respectively. Similarly, the study made by Sharma and Sharma (2024) who also achieved p value less than 0.05 while optimizing the process parameters for bioethnol production from ligno cellulosic waste.

Normally, a regression model having an R^2^ value > 0.9 is considered to have a very high correlation. The closer R^2^ (correlation coefficient) is to 1.0, the stronger the model and the better it predicts the response (Sharma and Sharma 2024).To check the fit of the model, the regression coefficient (in terms of coded) was calculated, and the variance analysis of the anticipated model of all R values (R^2^, R^2^ adj, and R^2^ pre) were used. The coefficient of determination of the R^2^ value for the model showed the best correlation between the predicted and experimental values. A lower *P*-value indicates greater interaction significance, and a higher R^2^ value shows a higher percentage of variation in the response of the 3D model. For alkali and acid pretreatments, the results of the ANOVA for cellulose, hemicelluloses, and lignin, were presented in supplementary tables (S1, S2, S3, S4, S5, and S6). The predicted 3D graph of RSM showed 92%, 91%, and 86% (alkali) and 76%, 75%, and 76% (acid), respectively. The R^2^ values (96%, 68%, and 84%) for cellulose, hemicellulose, and lignin showed that it is more suitable for acid pretreatment than alkali, according to the 3D model. The ANOVA results showed a strong correlation between the predicted and the experimental values. A study made by Samantaray et al. (2024b) investigated the role of the determining factors that showed the highest impact on the delignification content and the release of cellulose (mg g^−1^) and hemicellulose (mg g^−1^) during acid pretreatment. In their investigation, under optimal conditions, which included 60 min of incubation, 90 °C of temperature, 2 g of SC, and 2% (v/v) of acid concentration, 449 mg g^−1^ of cellulose was exposed in the case of diluted acid pretreatment. Following dilute acid pretreatment, Alavijeh et al. (2023) used corn stover as the lignocellulosic waste for the sustainable production of bioethanol, biodiesel, and biogas.

### Effect of ultrasonication pretreatment

Among various pretreatment methods, ultrasound pretreatment (US) enhances biomass solubilization through cavitation, generating extreme conditions (up to 5000 K, ~ 100 MPa), intense shear forces, and radicals (•OH, •O). Cavitation-induced effects include pore and crack formation, fiber separation, cellulose decrystallization, and lignin removal via cleavage of lignin-carbohydrate bonds (Luo et al. 2018; Bundhoo and Mohee 2018). In the present study, ultrasonication combined acid and alkali pretreatment was applied for the maximum delignification of CHB, and it was observed that there was an increase in delignification compared to the acid and alkali pretreatment. It resulted the maximum cellulose exposure of 706 mg g^−1^ and 690 mg g^−1^, respectively, with a power supply of 100 W at a temperature of 30 °C, 50% duty cycle was maintained in this state for 60 and 50 min, respectively. Keeping all the above constant parameters, ultrasonication-assisted alkali pretreatment showed the maximum release of hemicellulose of 165 mg g^−1^ and delignification of 22.2 mg g^−1^, and in ultrasonication-assisted dilute acid pretreatment, hemicellulose was obtained, i.e., 189 mg g^−1^, and lignin was removed up to 17.3 mg g^−1^. It was shown that the ultrasonication- assisted alkali pretreatment showed 28% higher delignification than the ultrasonication-assisted acid pretreatment. Through intensified heat and mass transfer, the ultrasonic energy improves the reactants and dissociation of products. C–C, C–H bond, H, and OH link breakage and formed macro radicals during pretreatment and forming cavitation, which induce the disruption of the ether bond of lignin. These results demonstrated that the depolymerization of lignin exhibits a significant advantage over its native structure. There is a breakdown of glycosidic bonds between cellulose and hemicellulose and a decrease in the carbohydrate content at higher ultrasonication times of 6–9 h (Rekha and Saravanathamizhan 2021). An increase in the sonication time increases the delignification from 40 to 60%. Modifications in the crystallinity index (CrI) values, the functional groups of the material, the increased surface porosity and area, and the decreased lignin all demonstrated the coagulation phenomenon of US-assisted alkaline pretreatment. The ultrasonication (US) pretreatment exhibited varying effects on total reducing sugar (TRS) production, highlighting the influence of temperature and amplitude. In all cases, the amounts of TRS increased over time, with higher amplitudes and temperatures enhancing the depolymerization efficiency of complex carbohydrates. Maximum sugar concentrations (605 mg g^−1^) were achieved after 60 min of treatment at 100% amplitude and 50 °C. Overall, a combination of higher temperature and longer treatment time ensured maximum solubilization. During ultrasonication alkali pretreatment enhanced cellulose and hemicellulose exposure up to 51 and 46% and delignification up to 49% (Table S7). In a similar type of study, Karanicola et al. (2021) demonstrated that 40% (w/w) of reducing sugars were extracted under ultrasound-assisted acid hydrolysis, using orange peel at 5.25% (w/v) and H_2_SO_4_ for 34 min at 100 °C. Likewise, Majumdar et al. (2018) achieved a maximum reducing sugar yield of 315 mg g^−1^ from orange peel pretreated at 121 °C and 15 psi for 30 min using 2.5% (v/v) sulphuric acid. Olguin-Maciel et al. (2024) investigated the ultrasonic pretreatment coupled with commercial enzymes and crude enzymes from a native fungus (*Trametes hirsuta Bm*-2) with a power of 70W at 42 kHz for 30 min at 45 °C, 60 °C, and 70 °C, which was used for delignification of corn fibre. The results showed that the pretreatment conditions do not affect enzymatic activity; rather, they lower the operating temperature, with 45 °C producing the optimum results.

### Optimization of different parameters for enhanced production of cellulase and xylanase

In the present study, several cellulolytic bacterial strains were isolated from cow dung samples, and the preliminary screening was done to evaluate their enzyme production ability. Further, the strains were subjected to morphological and biochemical identification. Based on the result, the cellulase-producing bacterial strain (CDB2) was identified as *Bacillus licheniformis.* For the xylanase enzyme previously isolated and identified, bacterial strain *Enterobacter asburiae* PQ396173 was used. Conditions were optimized for increased cellulase synthesis from the isolated bacterial strain which yield 9.3 ± 0.3 U mL^−1^ enzyme activity when 1% (v/v) of 24 h old inoculums was added to the medium (1% substrate, 1% beef extract, 0.5% K_2_HPO_4_.3H_2_O, 0.1% KH_2_PO_4_, 0.05% MgCl_2_ and 0.05% CaCl_2_ of pH 7.0 incubated at 40 °C temperature 36 h. In the present study, the cellulase activity of the isolated bacterial strain was comparatively higher than the activity of the bacterial strain studied by Lokapirnasari et al. (2015), i.e., 0.10 U mL^−1^. The findings of the study was at par with findings of Bhatia et al. (2017), who also reported a similar type of cellulase activity in a bacterial strain after 36 h of incubation. Like cellulase, xylanase production were also optimized under same parametric conditions which showed maximum activity i.e. 7.0 ± 0.36 U mL^−1^ after 24 h of incubation at 35 °C in modified Riviere’s medium (pH 9, 2% substrate, 1% meet extract, 0.5% KH_2_PO_4_, 0.5% (NH_4_)_2_SO_4_, 0.1% NaNO_3_, 0.1% CaCl_2_, 0.2% MgSO_4_, 0.15% FeSO_4_.7H_2_O, 0.1% MnCl_2_.4H_2_O.

### Optimization of enzymatic hydrolysis of pretreated CHB

**Fig. 3:**
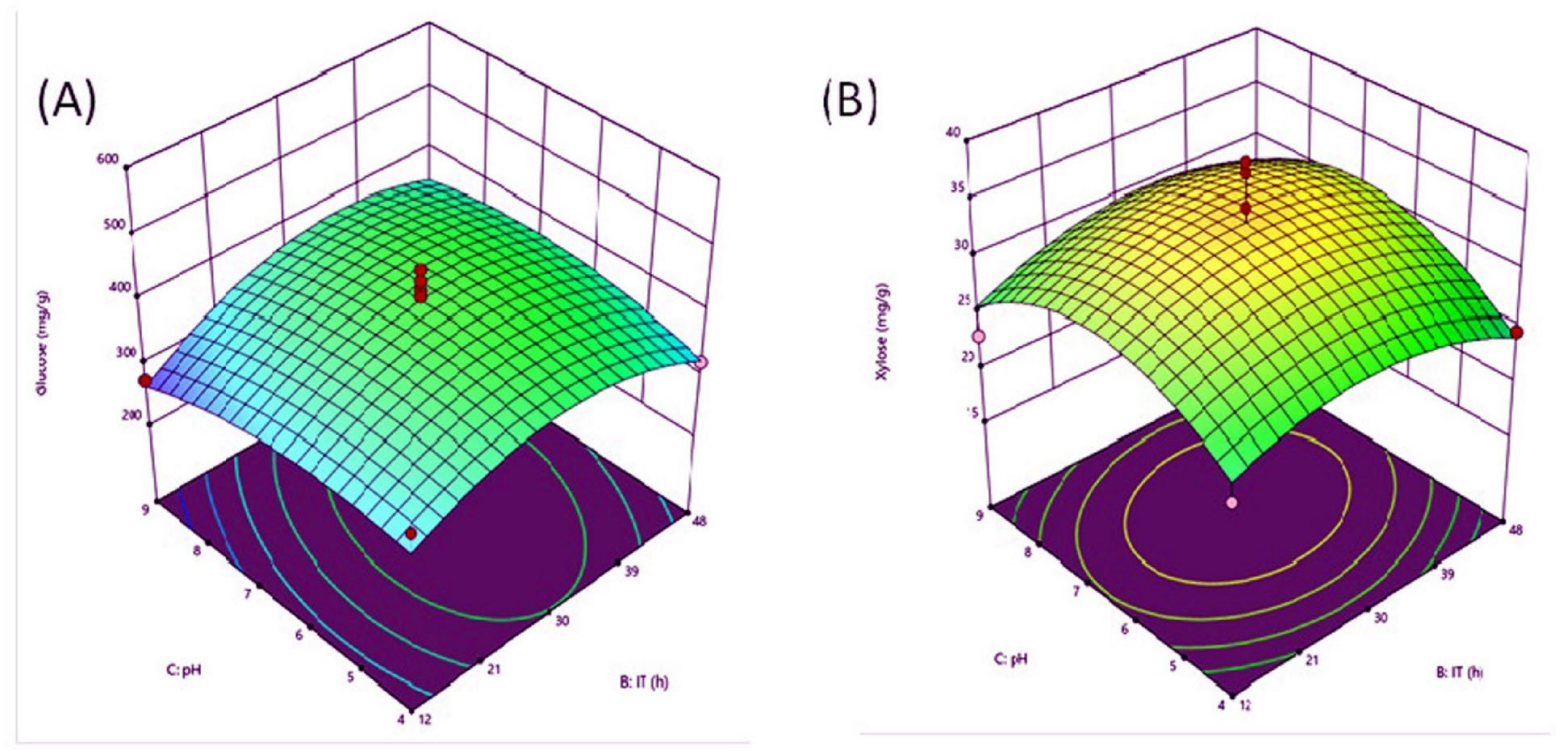
RSM graph showing effect of process parameters on the recovery of **A** Glucose, **B** Xylose

Based on the results of the pretreatment, the enzymatic hydrolysis experiment was designed and optimized using RSM. For enzymatic hydrolysis, both commercial enzymes (Celluclast 1.5 L (700 EGU and Viscozyme 13.4 FBG) and native cellulase and xylanase enzymes (2:1:3:3, v/v; U mL^−1^) from *Bacillus licheniformis* and *Enterobacter asburiae* PQ396173 were used. As a result, the parameters T (40 °C), IT (48 h), EC (U mL^−1^) (2:1:3:3), and pH (5.5) were taken into account, and the total glucose and xylose production from CHB was 558 and 47 mg g^−1^, respectively (Table 4). Among the four factors, it was found that pH and incubation time have the highest effects on glucose and xylose production (Fig. 3A and B). Moreover, it was found that the shorter incubation time produced lower yields of total sugar. Zhu et al. (2024) observed the greater effect of the parameters, including pH, enzyme loading, and temperature, on the enzymatic hydrolysis process of corn cobs. In this context, the cellulase activity is influenced primarily by temperature and pH. The optimal ranges for cellulase activity are typically 4–5.5 pH and 45–65 °C temperature (Yang et al. 2024). José et al. (2022) also optimized the enzymatic hydrolysis using RSM for maximizing the production of glucose (0.69 gL^−1^ h^−1^) and conversion of cellulose to glucose (90% w/w), as well as to xylose, hemicelluloses, and arabinose (44% w/w) with the help of commercial enzyme, i.e., Cellic Ctec2 (32 FPU g^−1^). Enzymolysis of cellulose involves three primary steps: the cellulase adsorbed on the cellulose surface, which hydrolyzes the cellulose to reduce glucose, and cellulase desorbed from the residues' surface (Zhu et al. 2024). Analysis of variance (ANOVA) is used to determine more than two different groups. The derived 3D model showed a higher *F*-value along with R^2^ values of 92% and 88% for xylose and glucose (Tables S8 and S9) correlation of *p*-values below or equal to 0.05, which indicated that the model was statistically significant (Hundie 2021).

**Table 4:** Enzymatic hydrolysis of pretreated Corn husk

Enzyme ratio (v/v) (mL) cellulase: xylanase	Cellulase (U/g biomass)	Xylanase (U/g biomass)	pH	Temp (°C)	Time (h)	Gluose (mg g^−1^)	Xylose (mg g^−1^)
3:1	1.75	0.44	4.8	45	24	271	20.2
3:2	1.40	0.70	5.5	50	24	458	40.0
3:3	1.16	0.88	5.5	45	48	280	24.6
2:1	1.55	0.58	5.5	45	36	196	22.3
2:2	1.16	0.87	6.2	45	48	456	32.4
2:3	0.93	1.05	5.5	50	48	354	24.7
1:1	1.16	0.87	5.5	50	36	324	22.7
1:3	0.58	1.31	6.2	45	36	499	43.4
1:2	0.77	1.16	4.8	45	48	255	17.6
**3:3**	1.16	0.87	5.5	40	48	558	47.0
3:2	1.40	0.70	4.8	40	36	301	23.0
3:1	1.74	0.43	4.8	50	36	425	37.8
2:2	1.16	0.87	4.8	45	36	368	32.6
1:3	0.58	1.31	5.5	40	24	241	11.2
2:1	1.55	0.58	6.2	45	36	296	23.5
3:3	1.16	0.87	5.5	45	24	240	28.8
3:2	1.40	0.70	4.8	45	36	305	29.3
3:1	1.74	0.43	6.2	45	24	440	38.7
2:2	1.16	0.87	5.5	45	48	370	25.0
2:3	0.93	1.05	5.5	40	36	426	30.0
2:1	1.55	0.58	5.5	45	24	50	36.4
3:1	1.74	0.43	5.5	40	36	277	21.0
3:2	1.40	0.70	6.2	40	36	437	46.3
3:3	1.16	0.87	5.5	45	36	348	24.0
1:2	0.77	1.16	5.5	45	36	315	27.0
1:3	0.58	1.31	6.2	50	36	234	18.2
1:1	1.16	0.87	5.5	50	36	265	27.6

### Saccharification using cellulase and xylanase cocktail

To assess the efficacy of cellulase and xylanase, the enzyme was purified entirely (> 80% purity). The crude extracellular extract was utilized in the case of isolated bacterial cellulase from *Bacillus licheniformis* (9.3 ± 0.3 U mL^−1^) and xylanase enzymes from *Enterobacter asburiae* PQ396173 (7 ± 0.36 U mL^−1^) were used**.** The enzyme activity was measured using beech wood xylan as the substrate under the conditions applied for the enzymatic hydrolysis of PBAB. Xylanases are mostly extracted from *Enterobacter asburiae* PQ396173 (6.89 ± 0.36 U mL^−1^) were used using xylan as a substrate**.** With the help of the bacterial enzyme (cellulase and xylanase) cocktail, the delignified corn husk biomass was saccharified. For better saccharification of CHB, various reaction conditions were optimised. Both enzymes were applied concurrently to the lignin-free biomass. For this experiment, different enzyme ratios of cellulase and xylanase concentrations (1:1; 1:2; 2:1, 3:1, v/v; U mL^−1^) were used. Once the reaction was completed, the maximum amount of reducing sugar (740 mg g^−1^ glucose and 54.6 mg g^−1^ xylose) was released when CHB was supplemented with a (v/v, U mL^−1^) 3:1 ratio of cellulase and xylanase respectively. It is possible that the reduced binding capacity of the substrate to the enzyme's active site and the saturation of the enzyme's active sites are the reasons why the yield of reducing sugars was lowest when both enzymes were given equal doses (v/v, U mL^−1^) (1:1). While estimating the reducing sugar from the pretreated CHB hydrolysate, glucose and xylose were taken as the standard sugars. An optimized combination ratio of (v/v, U mL^−1^) 3:3 was used for the final enzymatic hydrolysis. Our current study found that biomass loading (100 g), enzyme loading (v/v, U mL^−1^) (3:3), and incubation time of 96 h at 35 °C showed optimum enzymatic hydrolysis, releasing a total reducing sugar of 89.2 g L^−1^. It is evident that pretreatment is absolutely necessary to convert the biomass to sugars because the untreated biomass did not yield any reducing sugar even after enzymatic hydrolysis (Mankar et al. 2021).

In our investigation, we compared the effectiveness of isolated bacterial cellulase and xylanase enzymes with commercial enzymes. It was observed that the isolated enzyme mixture of cellulase and xylanase (v/v) (U mL^−1^) (3:3) converted maximum cellulose and hemicellulose to simple sugar i.e. 43.5 of glucose and 26.6 mg mL^−1^ of xylose, where isolated the single bacterial cellulase enzyme was able to convert cellulose to glucose i.e., 39.8 mg mL^−1^, and isolated xylanase was able to convert hemicellulose to xylose i.e. 21.3 mg mL^−1^. Meanwhile, the commercial cellulase and xylanase enzyme mixture cocktail (v/v) (U mL^−1^) (3:3) was able to convert CHB to simple sugar, i.e., 78.5 mg mL^−1^, but in the case of only cellulase, it was able to produce 44.8 mg mL^−1^ of glucose, whereas the xylanase was able produce 29.4 mg mL^−1^ xylan. The final enzyme dose was expressed in terms of units per gram of dry biomass (U/g biomass), and the corresponding values were calculated and are presented in Table 4.

For the enhancement of sugar conversion, all the commercial and isolated enzymes were mixed, and a cocktail was prepared at different proportions (v/v; U mL^−1^) (2:1:3:3). At the end, it was observed that after enzymatic hydrolysis using a cocktail, the conversion rate was high. After hydrolysis the total reducing sugar (89.2 mg mL^−1^) was estimated while cocktail cellulase was able to convert cellulose to glucose i.e. 53.8 mg mL^−1^ and cocktail xylanase was able to convert hemicellulose to xylose i.e. 34.4 mg mL^−1^ (Fig. 7). Actual enzyme activities were found to be more than the addition of individual enzymes. This proves that celluclast and accessory enzymes work in a synergistic manner. Our result is as per the finding of Ningthoujam et al. (2023) who made a comparison between isolated and commercial enzymes for hydrolysis of rice straw biomass for bioethanol production. Similarly in another study of Nath et al. (2021) found the highest TRS using commercial and native cellulase and xylanase enzyme cocktail while scarifying sugar cane bagasse and sorghum straw respectively.

The calculated enzyme loading demonstrated that the applied cellulase and xylanase activities correspond to a fixed dosage relative to substrate mass, ensuring uniform enzyme–substrate interaction conditions. Such normalization is critical because volumetric enzyme addition alone does not account for variations in substrate concentration, which can significantly influence hydrolysis efficiency (Mezule et al. 2019; Kumar and Verma 2024). The biomass-normalized enzyme loading enabled better interpretation of saccharification results, showing a direct relationship between enzyme dosage and sugar yield. Treatments with optimized enzyme loading exhibited higher glucose and xylose release, indicating improved hydrolytic performance. This also facilitates direct comparison with previously published studies, where enzyme loading is typically reported in U/g biomass or FPU/g substrate (Kristensen et al. 2009; Luo et al. 2019).

### Surface characterization of untreated and pretreated cornhusk biomass

**Fig. 4:**
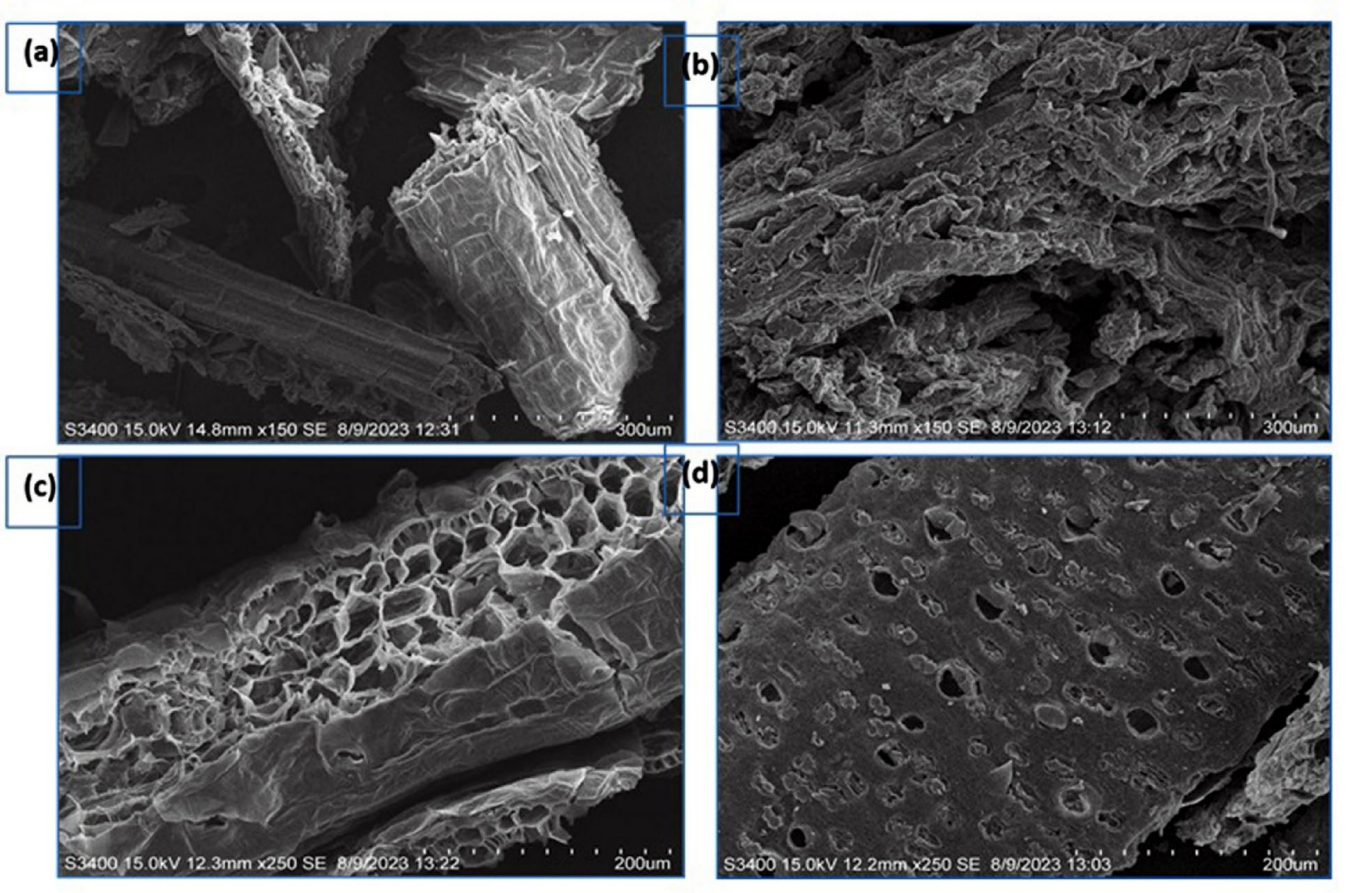
SEM Analysis of raw and pretreated corn husk biomass **a** raw, **b** acid pretreated, **c** UA-NaOH pretreated and **d** enzymatic hydrolysed CHB

Following pretreatment and enzymatic hydrolysis, the hydrolyzed CHB was subjected to SEM, FTIR, and XRD analysis to predict the structural changes in the substrate. It is visible that the pretreated CHB displayed a disrupted internal surface, but the raw CHB displayed a closed, solid structure with a uniform and smooth surface (Fig. 4a and b). The ultrasonication process mainly eliminated the lignin and hemicellulose in CHB. Following ultrasonication pretreatment, the smooth and uniform surface of raw CHB had many micropores, which enhanced the available surface area of CHB. Compared to the raw CHB, the outside layer of the pretreated CHB displayed several observable changes. Ultrasonication-assisted alkali pretreated biomass resulted in apparent fiber degradation and breakage, as well as some layering and scaling (Fig. 4c). In comparison to the ultrasonication assisted alkali (UA-NaOH) pretreatment sample, the cellulose fiber surface exhibited notable cracks, fractures, and dissolution troughs after treatment with UA-H_2_SO_4_ (Fig. 4d). The increased disintegration observed within the biomass structures may be attributed to the enhanced effects of alkali in conjunction with elevated temperature and pressure thereby rendering it the most advantageous option for enzymatic hydrolysis. The results indicate that UA-NaOH pretreatment effectively facilitated the removal of a substantial quantity of lignin.

**Fig. 5:**
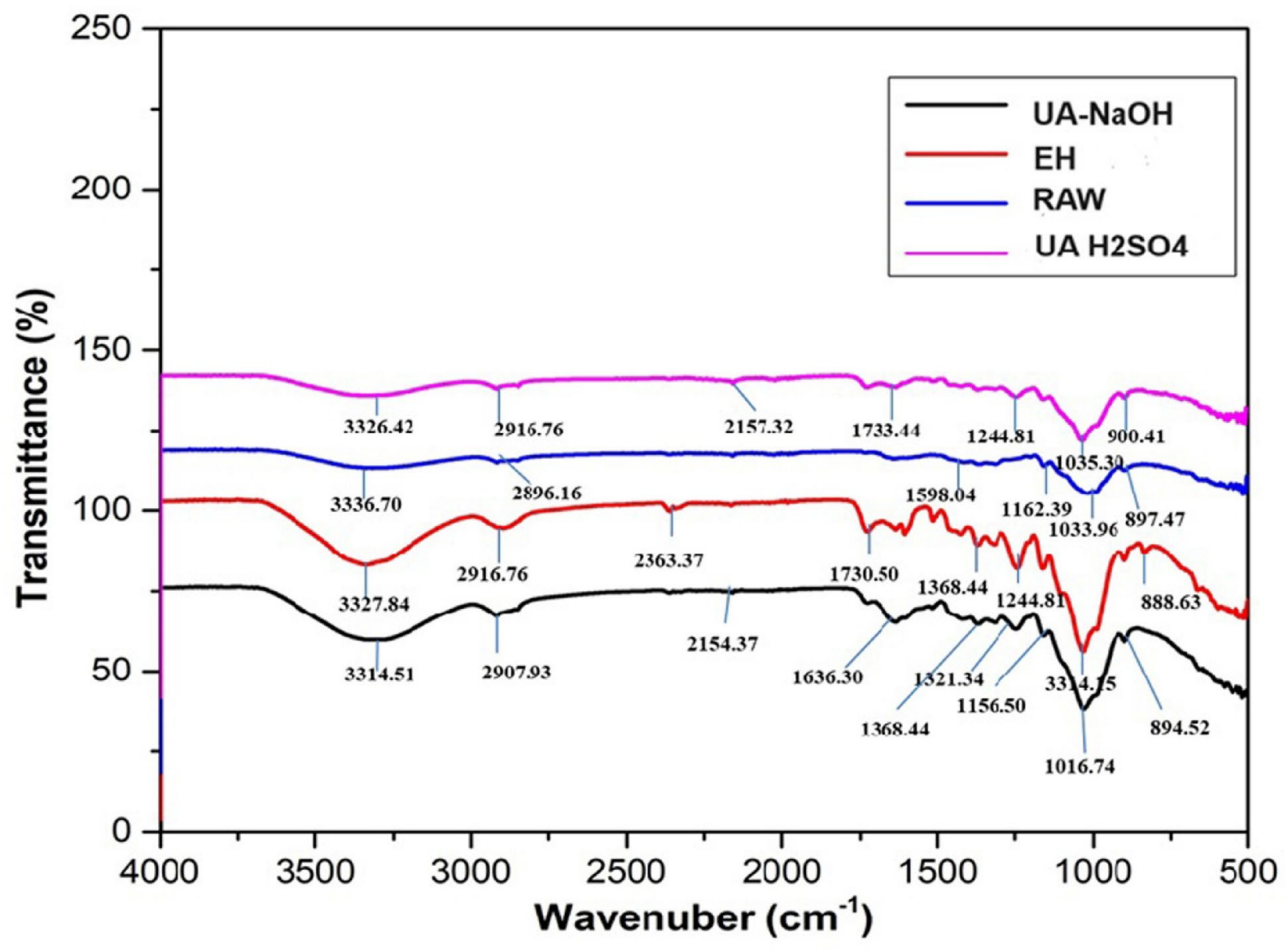
FTIR Analysis of raw and pretreated corn husk biomass

FT-IR spectra are used to analyze the structural changes of raw biomass as well as UA-H_2_SO_4_, UA-NaOH, acid, alkali, and enzymatically hydrolyzed CHB variations (Fig. 5). A broad absorption band in the region of 3200–3600 cm^−1^ was attributed to O–H stretching vibrations, while peaks near 2900 cm^−1^ correspond to C–H stretching vibrations. The amount of cellulose in UA-H_2_SO_4_ pretreatment samples was revealed by the absorption peaks, which measured 3314.1 cm^−1^. The lignin and hemicellulose are released when the lignin carbohydrate complex aromatic linkages are broken down by ultrasonication pretreatment of CHB. Sonication creates a particular physicochemical environment that changes the biomass's surface by mechano-acoustic cavitation and asymmetric micro-bubble formation and breakdown, leading to biomass disruption. When the outer barrier of lignin breaks down, the amorphous hemicellulose area also breaks down. As seen in Fig. 5, the cyclic linkages and phenol hydroxyl groups of lignocelluloses are represented by 1156.5, 1016.7, 1321.3, and 1368.4 cm^−1^, respectively. This suggests ultrasound may facilitate the breakage of α and β-aromatic bonds in lignin. The lignin bands at the peak of 1636 cm^−1^ show the response to vibrations of phenol hydroxyl groups, C–H deformation, and C–C alkene aromatic structure. The C-H and CH_2_ stretching of aromatic groups in lignin is indicated by the absorption band at 2907.9, 2849.1 cm^−1^. In addition to bubbling, the breakage of the C–C and C–H bonds results in macro-radicals in the medium, promoting the depolymerization of lignocellulosic biomass. These hemicellulose group peaks, which correspond to ketone or aldehyde C–O stretching and C–O stretching of acetyl groups, were detected at approximately 1730.5 and 1244.8 cm^−1^. Peaks formed about 1633.4 cm^−1^ in processed biomass, indicating that the hemicellulose was exposed due to the breaking of lignin and hemicellulose interactions. The treatments broke the ether linkages, exposing more cellulose surface to the release of sugar. The intermolecular and intramolecular hydrogen bonding in cellulose is shown by the peaks at 3417 and 3414 cm^−1^. The β-glycosidic bond with characteristic peak of cellulose at 894.5 and 888.6 cm^−1^ shows that the sample has broken down cellulose following pretreatment.

**Fig. 6:**
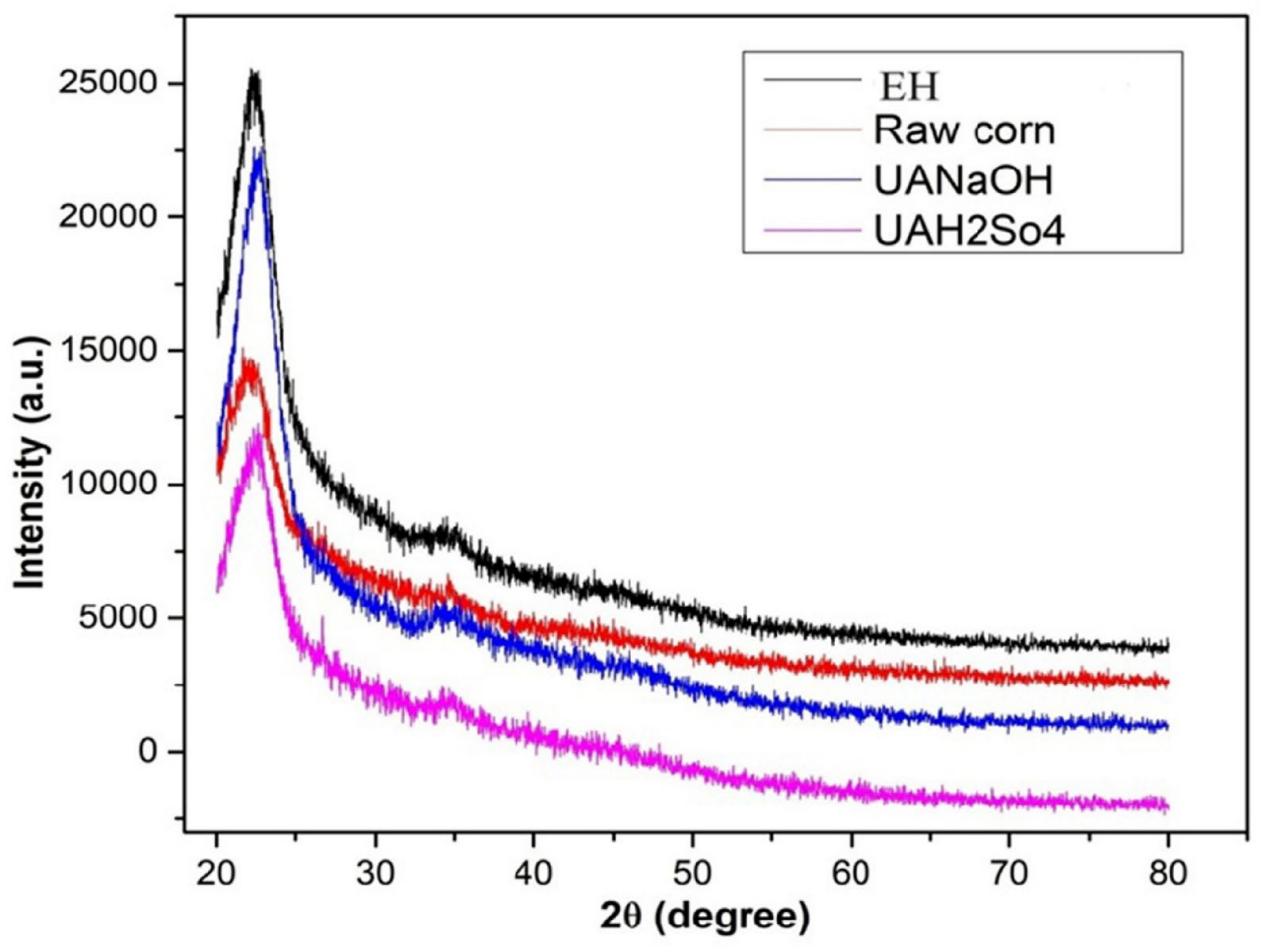
XRD Analysis of raw and pretreated corn husk biomass

The XRD method was used to evaluate the structural changes. The crystallinity index (CI) is based on the ratio of crystalline material in biomass (Fig. 6). The untreated CHB had a crystallinity index (CrI) of 20–26%, whereas UA-H_2_SO_4_, UA-NaOH pretreated hydrolysate CHB exhibits crystallinity index (CrI) values of 26–45%. CrI dropped from 51 to 30 and 23% for UA-NaOH and UA-H_2_SO_4_ pretreatment, respectively, which may be the outcome of crystalline cellulose transforming into its amorphous form, resulting in the biodegradable qualities of cellulose (Fig. 6). The intensity peak was found in 23% after enzymatic hydrolysis, which indicates the decrease in the crystalline portions of the substrate. The lignin breakdown that exposed the internal cellulose surfaces enhanced the quantity of crystalline cellulose. The crystallinity of biomass significantly increased to 30%, resulting in the removal of lignin and amorphous hemicellulose. According to this study, the crystallinity increases more when treated with UA- NaOH than with other pretreatments (Fig. 6).

**Fig. 7:**
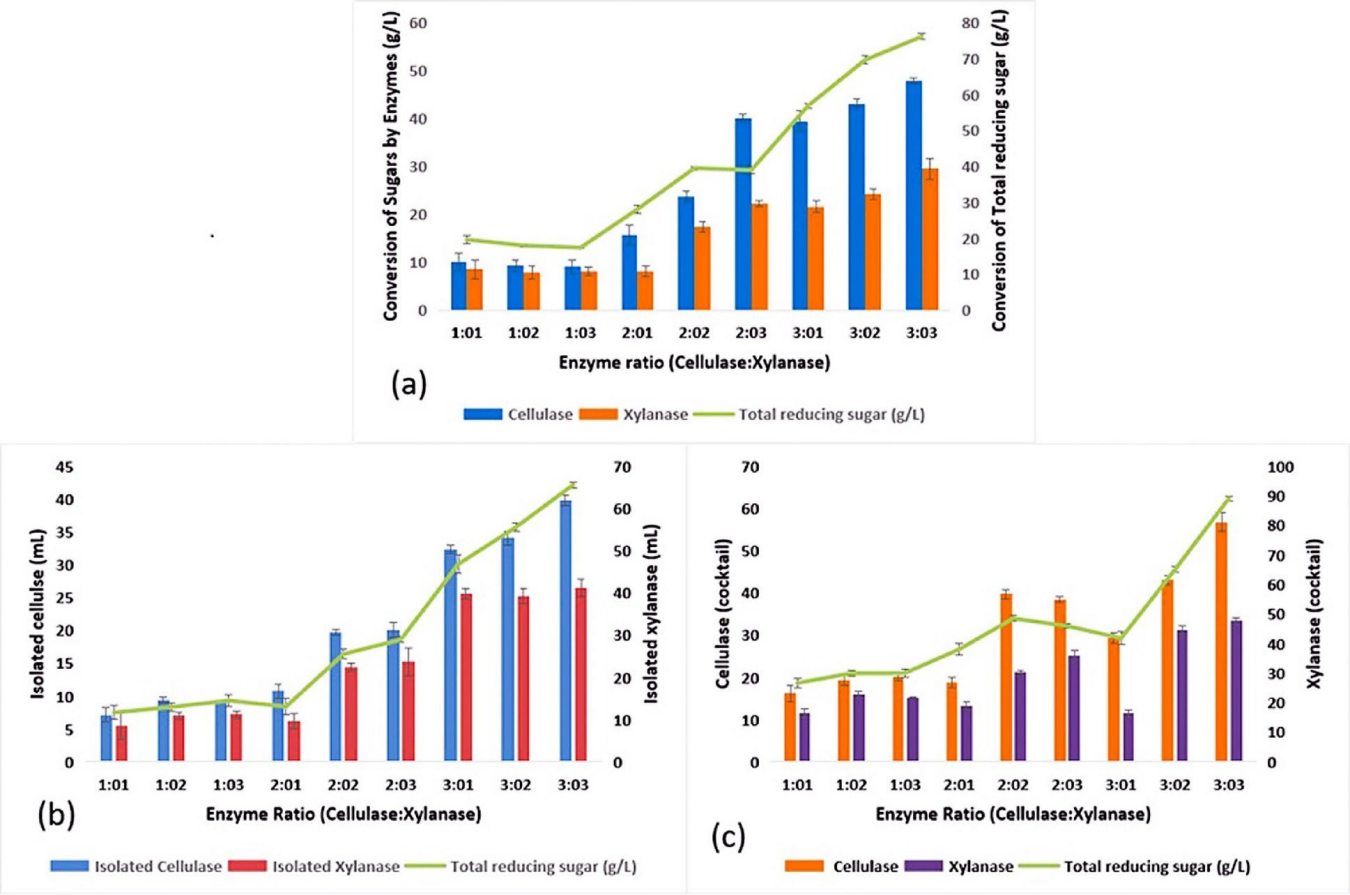
Sugar utilization **a** by commercial enzymes, **b** by isolated enzymes and **c** by cocktail of both isolated and commercial enzymes of hydrolyzed corn husk biomass

### Simultaneous saccharification and co-culture fermentation

**Fig. 8:**
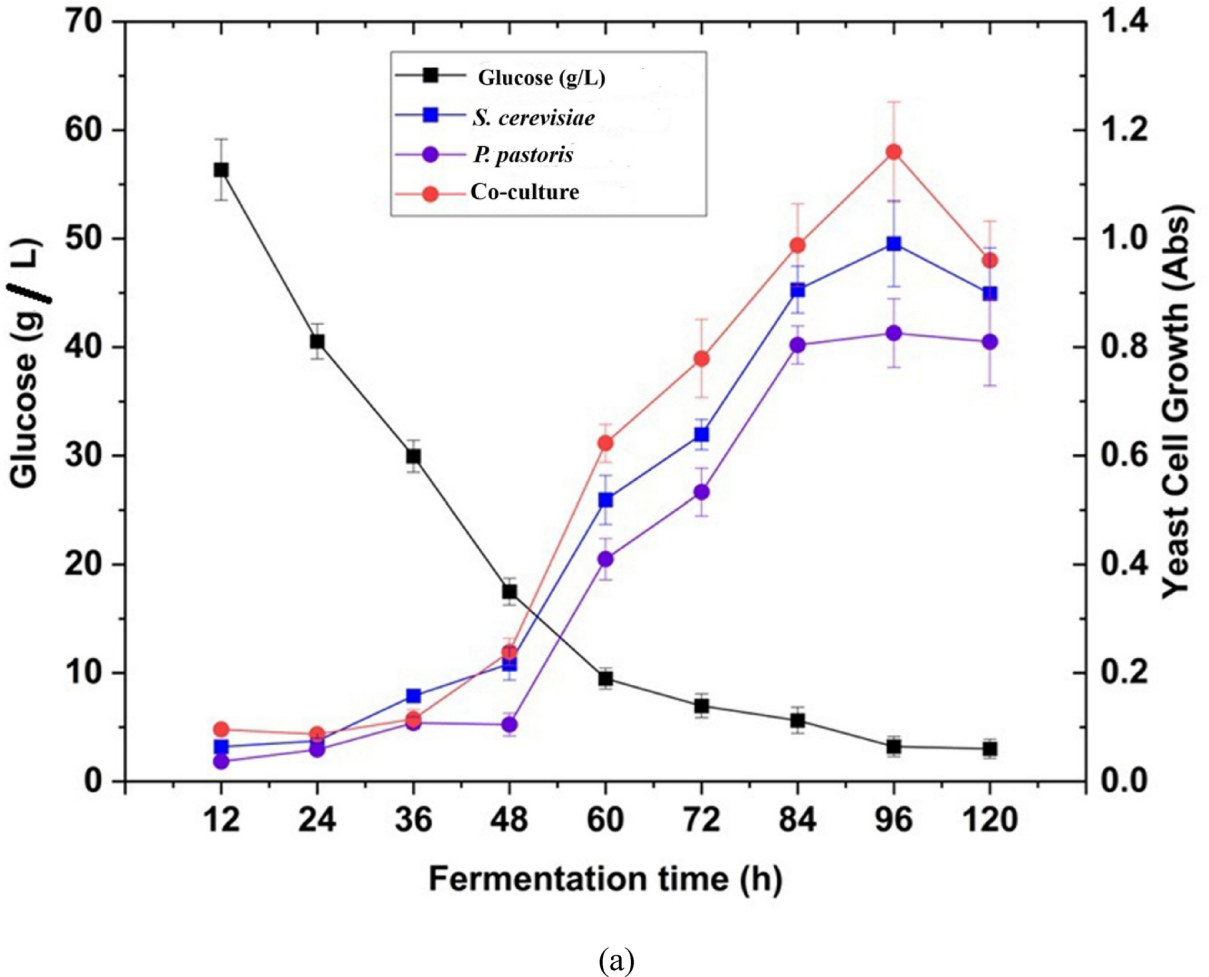

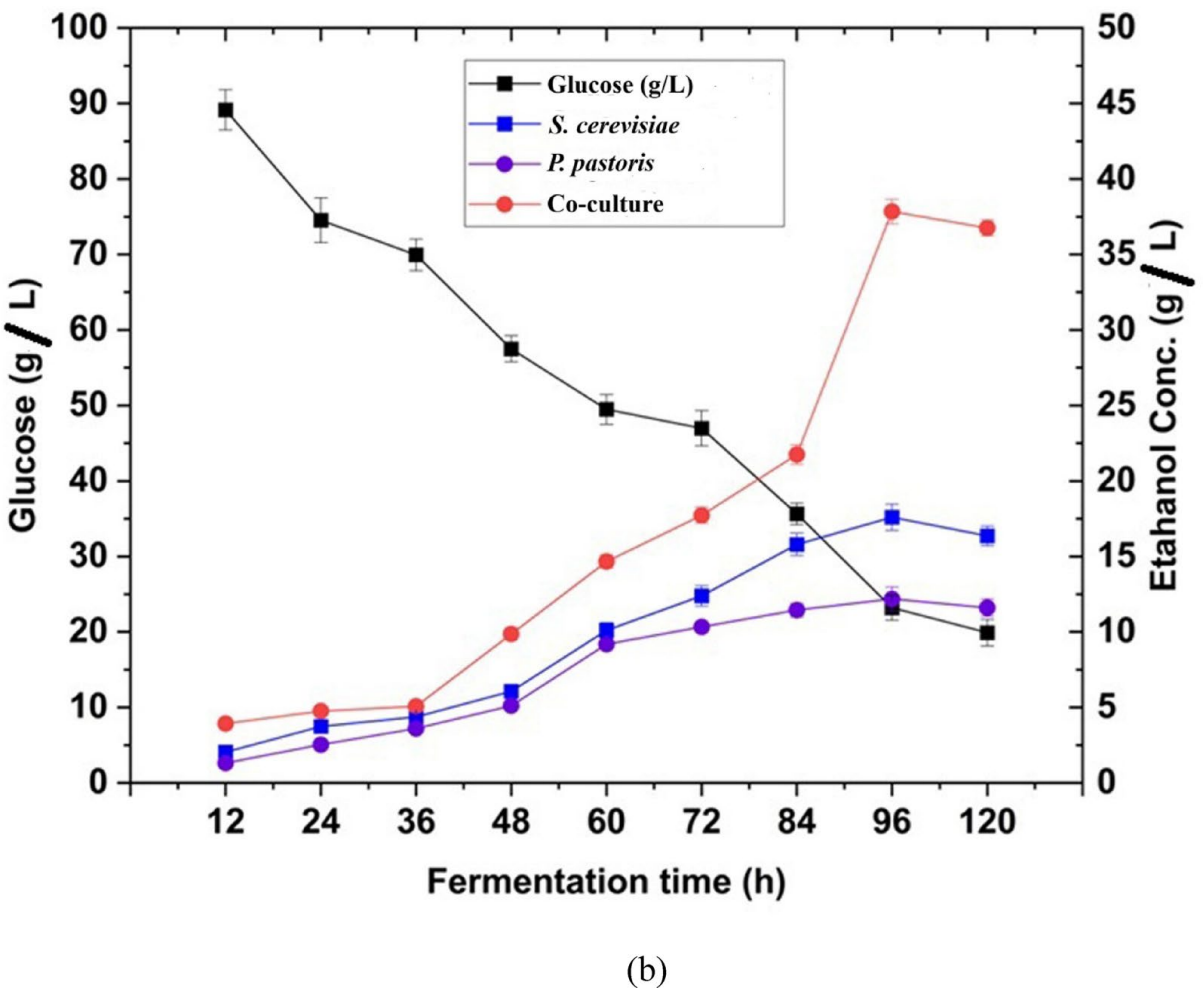
**a** Changes of yeast cell growth and progression of sugar utilization by fermentation of hydrolyzed corn husk. **b** Progression of sugar utilization and bioethanol production by fermentation of hydrolyzed corn husk

As described in figure 7, the total reducing sugar yield obtained after enzymatic hydrolysis using enzyme cocktail reached 89.2 mg mL⁻¹, indicating effective saccharification. Further, simultaneous saccharification and co-culture fermentation (SHCF) techniques are employed for the conversion of hexose and pentose sugars (glucose, xylose) found in the hydrolyzed CHB using two well-studied yeast strains that are *S. cerevisiae* (MTCC 170) and *P. pastoris* (MTCC34). Figure 8a and b represent the relationship between two yeast strains, the utilization of sugar at different time intervals, and the production of ethanol accordingly. After 96 h of incubation, approximately 85% of glucose and 80% of xylose is converted into ethanol. The hydrolysates from the diluted acid and alkaline hydrolysis were fermented using *S. cerevisiae* and *P. pastoris* at 30 °C, with 200 rpm for a 12 h fermentation period (12–96 h). Untreated CHB had an initial cellulose content of 110 mg g^−1^ before pretreatment. In contrast, the highest cellulose content of 706 mg g^−1^ was determined following the ultrasonication-assisted alkali pretreatment. The fermentation rate increases with the increase in sugar concentration; however, at a certain point, the fermentation rate will remain constant whether the sugar level increases. This is because the amount of sugar being used is more than what the microbial cells can convert (Ding et al. 2023). The highest rate of ethanol production occurs at sugar concentrations of 100 g L^−1^. The initial sugar concentration is also a significant factor in the ethanol production. In batch fermentation, a higher initial sugar concentration can boost ethanol yield and productivity, which requires maximum time for fermentation and is effective to recover (Zabed et al. 2014). Varize et al. (2022) also reported that *S. cerevisiae* has a maximum production capacity of 14% ethanol. The growth rate of the yeast strain in the fermentation medium is inversely proportional to the microbial content, and it increases as the concentration of sugar hydrolysate decreases. The fermentation process took place within 96 h (Fig. 8a). Both yeast strains consumed sugars as a nutrient and converted them to ethanol under anaerobic conditions. The yeast cell undergoes several physiological changes during fermentation. As the fermentation progresses, the yeasts utilize all those nutrients and reduce the amount of sugar. Both strains (*S. cerevisiae* and *P. pastoris*) can convert sugar to ethanol separately at different time intervals in monoculture. It was quantified that the conversion of ethanol was 2.04–17.6 g L^−1^ in monoculture of *S. cerevisiae* and 1.3–12.2 g L^−1^ in monoculture of *P. pastoris*, respectively. In the co-culture, both the yeast strains were able to convert glucose to ethanol from 12 to 96 h (4.0–26.8 g L^−1^), which is comparatively higher than the monoculture (Fig. 8b). Further, the cocktail enzyme hydrolysates yielded a maximum ethanol of 37.3 g L^−1^ at 96 h incubation. After that, there is a gradual decline in the production of ethanol due to 91% of reducing sugar consumption at 96 h of incubation time. However, the decrease of ethanol concentration beyond 96 h implied that the stationary phase has already passed for the co-culture yeast cell in the fermentation medium and production of other secondary metabolites. A similar study conducted by Sharma et al. (2018) reported that maximum ethanol production (6.24%, v/v) was achieved through simultaneous saccharification and fermentation (SSF) using a co-culture strategy on pretreated corn husk after 24 h of incubation. In another study, Sharma et al. (2021), who reported the production of 4.1 g L^−1^ bioethanol from wheat straw using yeast co-culture fermentation after 96 h of incubation. In another study, Selvakumar et al. (2022) recorded 24.6 mg mL^−1^ of bioethanol production from corn cob using yeast co-culture fermentation technique up to 96 h of incubation. The present finding is also supports the finding of Behl et al. (2023), who reported 26.6 ± 0.46 g/kg bioethanol production from corn stalk using the co-fermentation by applying yeasts like *S. cerevisiae* and *K. marxianus* MTCC 1498. Previous research indicates that ethanol production is relatively higher in co-culture fermentation than in monoculture (Nuwamanya et al. 2012).

Xylose, a pentose sugar, can be converted to biofuel by some yeast species, but *S. cerevisiae* can't do this conversion. Utilizing a microorganism capable of fermenting both pentose (C5-xylose) and hexose (C6-glucose) sugars, as opposed to *S. cerevisiae*, may enhance ethanol yield from the hydrolysate. Accordingly, *P. pastoris* yeasts were employed in conjunction with *S. cerevisiae* for ethanol production. Similar findings by Su et al. (2023) suggested that the maximum glucose and xylose concentration was obtained at an optimum hydrolysis of pretreated poplar in comparison with corn stover at 96 h of incubation. In another study, Chen et al (2021) reported 30.9 g L^−1^ of glucose while valorising the sorghum straw for bioethnol production (19.2 g L^−1^). When *S. cerevisiae* (hexose fermenting yeasts), coupled with *Pichia fermentans* and *Pichia stipitis* (pentose fermenting yeasts) in a co-culture fermentation, they effectively utilize sugars (hexose and pentose) to ethanol (Karagöz and Özkan 2014). The outcome of the co-culture techniques must be compared to those of the *S. cerevisiae* or *P. pastoris* monocultures individually to assess their performance more accurately. In monoculture fermentation of hexose fermenting yeast, *S. cerevisiae* could not utilize pentose sugars like xylose, arabinose etc., as expected. In contrast, the monoculture of *P. pastoris* has little potential to utilize both pentose and hexose sugar molecules in fermentation techniques. Improving the performance ability of *S. cerevisiae* may be possible by adaptive evolution in the fermentation industry, as it was coupled with *P. pastoris*. Additionally, Rojas-Chamorro et al. (2020) found that when *S. stipitis* and *S. cerevisiae* were co cultured in brewer's spent grains (BSG) hydrolysates, sugar conversion was improved compared to *E. coli* in the monoculture. However, Farias and Maugeri Filho (2019) found that when *S. passalidarum* and *S. cerevisiae* were fermented in co-culture and also separately in monoculture. This was likely because oxygen was limited, and hexose-fermenting species accumulated ethanol more quickly.

Table S10 demonstrate the comparative study of the ethanol yield, pretreatment severity, enzyme loading, and fermentation strategy of different agro wastes for bioethnaol production of the previous and present study. In the present study, the ethanol yield of 37.3 g L^−1^ from hydrolyzed corn husk which are in agreement with the earlier reported data. The novelty of the present research lies in the fact that the isolated cellulase and xylanase enzyme from native bacteria could able to show higher enzyme activity compared to other bacterial enzyme which was used by other researchers for the saccharification of different lignocellulosic wastes. In a similar type of research, corncob was hydrolyzed using a cocktail of three different enzymes: a cellulase from *Actinobacillus* sp. with an activity of 0.032 ± 0.007 U mL^−1^, a xylanase from *Bacillus* sp. PC-01 with an activity of 0.142 ± 0.007 U mL^−1^, and a xylose isomerase from *S. griseus* with an activity of 0.088 ± 0.001 U mL^−1^ (Asmarani et al. 2020).

Using composition analyses after each step, we estimated an overall mass balance for bio ethanol production process, including the pretreatment, enzymatic hydrolysis, and fermentation steps (Table 5). CHB, after pretreatment, can be successfully converted to ethanol by the SSCF process. After pretreatment, 89.2 g of total reducing sugar were estimated, which is not too far off 100 g raw material. From the enzymatic hydrolysis step, 49.6 g of glucose and 23.5 g of xylose were obtained per 100 g of pretreated CHB, when the cocktail of commercial and native cellulase and xylanase (2:1:3:3, v/v; U mL^−1^) per gram CHB was used.

**Table 5:** Estimation of mass balance (g L^−1^) and conversion rate (%) of biomass to ethanol of pretreated and hydrolysed corn husk biomass

Methods	Output (components)	Mass balance of biomass before conversion (g L^−1^)	Mass balance of biomass after conversion (g L^−1^)	Conversion rate (%)	Ethanol production (g L^−1^)	Ethanol productivity (g L^−1^ h^−1^)
Pretreatment	Pretreated CHB	100	89.2	10.8	–	–
Enzymatic hydrolysis	Hydrolysates	89.2	54.6	45.4	–	–
Fermentation	Fermentative products	54.6	13.7	86.3	37.3	**0.389**

Fermentation of the hydrolysates by *Saccharomyces cerevisiae* and *Pichia pastoris* resulted in 37.3 g L^−1^ ethanol after 96 h, equivalent to 80% of the maximum theoretical yield (based on the amount of glucose in raw material). Using native microbial enzymes reduces costs associated with commercial enzymes, making the process more economically viable. Techno-economic assessments suggest that employing internally generated enzymes for saccharification, as implemented in this study, can substantially improve the economic feasibility of biorefineries. In second-generation (2G) ethanol production, the cost of commercial enzymatic cocktails constitutes a major economic constraint, accounting for approximately 43% of the overall production cost (Verma et al. 2021). The use of low-cost enzymatic formulations, such as those developed in the present work, therefore represents an effective approach to lowering processing costs. Additionally, integrating in-house enzyme production into biomass conversion processes promotes resource optimization and supports circular economy concepts, thereby enhancing the sustainability of lignocellulosic bioethanol production. In a similar type of study, Ariaeenejad et al. (2023) achieved a bioethanol concentration of 51.5 g L^−1^ using a bifunctional xylanase/β-glucosidase enzyme. The study concluded that ethanol concentration of 37.3 g L^−1^, having ethanol productivity of 0.389 g L^−1^ h^−1^ and conversion efficiency of 86% were obtained at 96 h incubation from corn husk is similar with the study of Punia and Kumar (2025) who reported ethanol concentration of 16.8 g L^−1^, with ethanol productivity of 0.26 g L^−1^ h^−1^ and conversion rate of 75% while investing sweet sorghum straw.

#### HPLC analysis of bioethanol

**Fig. 9:**
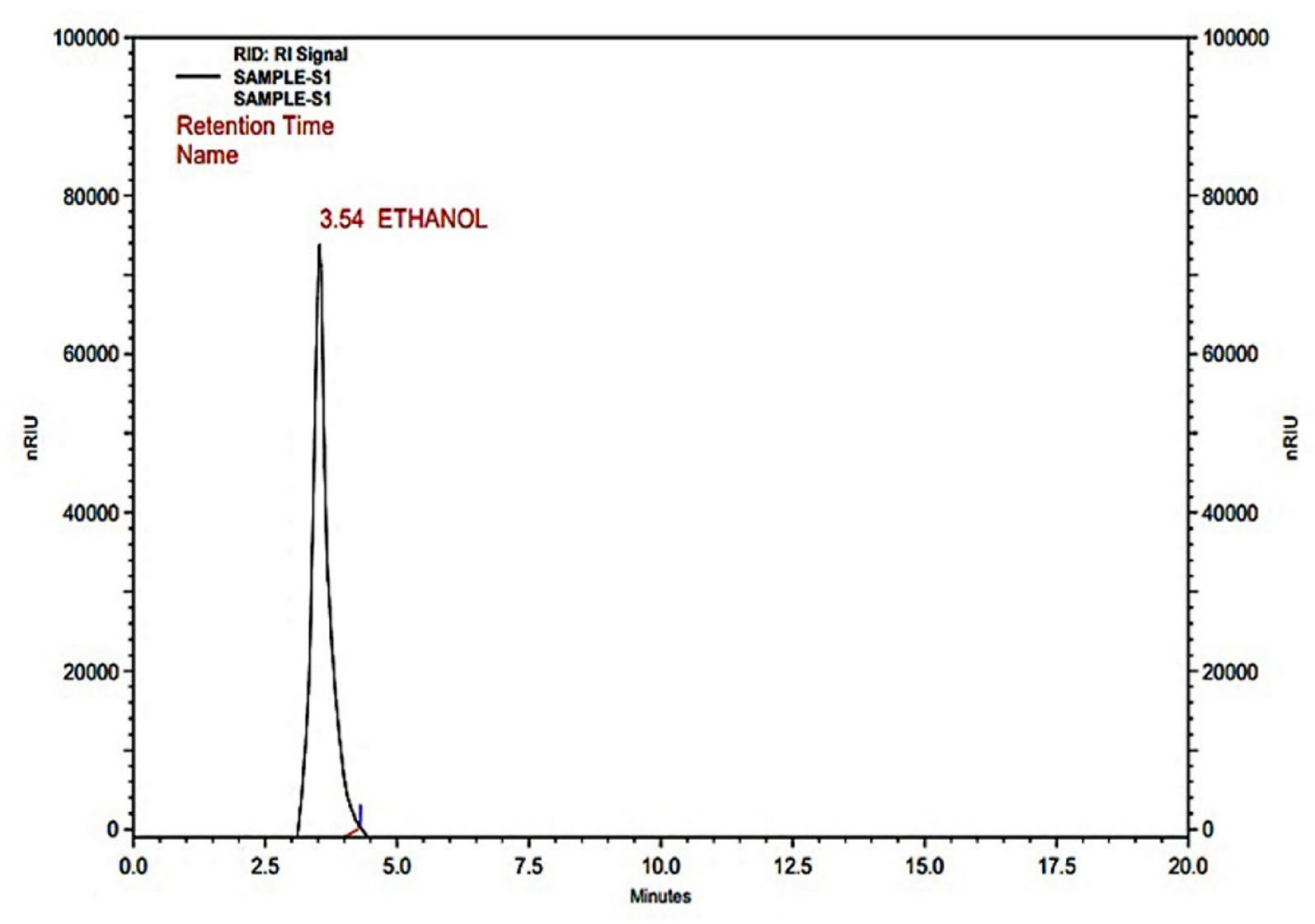
HPLC chromatogram showing the peak of Ethanol after distillation of fermented CHB

After the fermentation, the samples were distilled and purified to exclude the impurities of water and substances. Both qualitatively and quantitatively, the distilled bioethanol was evaluated using the HPLC technique. The peak was observed at a retention time (RT) of 3.54 min with a peak area of 1.73 × 10^8^, similar to the ethanol standard. The HPLC result revealed that the concentration of bioethanol was 26.8 g L^−1^ in the fermented broth using commercial enzyme treated corn husk hydrolysate. While the biomass hydrolysed with cocktail of isolated and commercial enzyme mixture yields 37.3 g L^−1^ of bioethanol (Fig. 9). Production of bioethanol and other organic chemicals of sugar hydrolysate from lignocellulosic wastes was previously quantified and characterized by researchers using HPLC (Valladares-Diestra et al. 2022; Ningthoujam et al. 2023). Rico et al. (2023) investigated the possibility of melon peels producing maximum bioethanol (56.2 g L^−1^), as measured by HPLC analysis using SSF techniques.

## Conclusion

The present study demonstrates the potential of corn husk biomass as a valuable resource for production of bioethanol, introducing an environmentally sustainable solution to manage agricultural disposal while addressing the growing energy demands. During UA-alkali pretreatment, the maximum lignin removal reached 35% and 39% hemicellulose, with 48% cellulose recovery respectively, in addition the developed enzyme cocktail of native and commercial enzymes, yielded total reducing sugar of 740 mg g^−1^ glucose and 54.6 mg g^−1^ xylose allowing for efficient fermentation using co-culture and monoculture techniques. The bioethanol concentration of 26.8 g L^−1^ at 96 h of incubation highlighted the utilization of corn husk waste as a renewable energy source. Further, using hydrolysate formed by a commercial and bacterial isolated enzyme cocktail exhibited a higher bioethanol concentration of 37.3 g L^−1^ at 96 h incubation. Studies have shown that combining enzymes play a synergistic role with different specificity enhances biomass degradation and bioethanol yield. This work also highlights the importance of ultrasono assisted alkali pretreatment along with use of native and commercial enzyme cocktail which removes a substantial amount of TRS (89.2 g) from lignin-free corn husk biomass loading of 100 g L^−1^ for maximizing the bioethanol production which is eco-friendly and cost-effective. However, further investigations into process optimization, economic feasibility, and environmental impact are essential to facilitate commercial scalability.

## Supplementary Information

Supplementary Material 1

## Data Availability

Data available upon request.
