# Supplementary material for: Optimization of pretreatment and enzymatic hydrolysis using commercial and isolated bacterial enzyme cocktail for bioethanol production from corn husk through yeast co-culture batch fermentation
Source: Bioresour Bioprocess. 2026 May 13;13(1):69. doi: 10.1186/s40643-026-01062-z (PMC13172165; doi:10.1186/s40643-026-01062-z)
Supplement: Supplementary file 1 — Supplementary Material 1 [file 40643_2026_1062_MOESM1_ESM.docx]

**Table S1.** ANOVA for the regression equation of cellulose production of NaOH pretreated corn husk biomass

| **Source** | **Sum of Squares** | **df** | **Mean Square** | **F-value** | **p-value** |  |
| --- | --- | --- | --- | --- | --- | --- |
| Model | 54035.7 | 14 | 3859.7 | 13.2 | < 0.0001 | significant |
| A-SC | 12554.9 | 1 | 12555 | 42.9 | < 0.0001 |  |
| B-ALK | 959.8 | 1 | 959.8 | 3.3 | 0.089 |  |
| C-Temp | 1351.2 | 1 | 1351.2 | 4.6 | 0.047 |  |
| D-IT | 1617.2 | 1 | 1617.2 | 5.5 | 0.031 |  |
| AB | 4120.5 | 1 | 4120.5 | 14.0 | 0.002 |  |
| AC | 3145.1 | 1 | 3145.1 | 10.7 | 0.004 |  |
| AD | 233.6 | 1 | 233.6 | 0.797 | 0.385 |  |
| BC | 1555.4 | 1 | 1555.4 | 5.31 | 0.034 |  |
| BD | 4340.8 | 1 | 4340.8 | 14.8 | 0.001 |  |
| CD | 499.9 | 1 | 499.9 | 1.71 | 0.209 |  |
| A² | 9791.4 | 1 | 9791.4 | 33.4 | < 0.0001 |  |
| B² | 6401.7 | 1 | 6401.7 | 21.9 | 0.0003 |  |
| C² | 2487.6 | 1 | 2487.6 | 8.5 | 0.01 |  |
| D² | 389.7 | 1 | 389.7 | 1.33 | 0.265 |  |
| Residual | 4686 | 16 | 292.8 |  |  |  |
| Lack of Fit | 4617.4 | 8 | 577.1 | 67.3 | < 0.0001 | significant |
| Pure Error | 68.5 | 8 | 8.57 |  |  |  |
| Cor Total | 58721.6 | 30 |  |  |  |  |

| **Std. Dev.** | **Mean** | **C.V. %** | **R²** | **Adjusted R²** | **Predicted R²** | **Adeq Precision** |
| --- | --- | --- | --- | --- | --- | --- |
| 17.1 | 432.2 | 3.96 | 0.920 | 0.85 | 0.315 | 16.9 |

**Table S2.** ANOVA for the regression equation of hemicellulose production of NaOH pretreated corn husk biomass

| **Source** | **Sum of Squares** | **df** | **Mean Square** | **F-value** | **p-value** |  |
| --- | --- | --- | --- | --- | --- | --- |
| Model | 2821.9 | 10 | 282.2 | 19.1 | < 0.0001 | significant |
| A-SC | 1.49 | 1 | 1.49 | 0.101 | 0.753 |  |
| B-ALK | 74.8 | 1 | 74.8 | 5.08 | 0.035 |  |
| C-Temp | 357.6 | 1 | 357.6 | 24.2 | < 0.0001 |  |
| D-IT | 83.3 | 1 | 83.3 | 5.66 | 0.027 |  |
| AB | 12.8 | 1 | 12.8 | 0.869 | 0.362 |  |
| AC | 635.4 | 1 | 635.4 | 43.1 | < 0.0001 |  |
| AD | 62.0 | 1 | 62.0 | 4.21 | 0.053 |  |
| BC | 154.7 | 1 | 154.7 | 10.5 | 0.004 |  |
| BD | 5.4 | 1 | 5.4 | 0.366 | 0.552 |  |
| CD | 565.5 | 1 | 565.5 | 38.4 | < 0.0001 |  |
| Residual | 294.6 | 20 | 14.7 |  |  |  |
| Lack of Fit | 102.9 | 12 | 8.58 | 0.358 | 0.946 | not significant |
| Pure Error | 191.6 | 8 | 23.9 |  |  |  |
| Cor Total | 3116.5 | 30 |  |  |  |  |

| **Std. Dev.** | **Mean** | **C.V. %** | **R²** | **Adjusted R²** | **Predicted R²** | **Adeq Precision** |
| --- | --- | --- | --- | --- | --- | --- |
| 3.84 | 97.8 | 3.93 | 0.905 | 0.858 | 0.789 | 17.6 |

**Table S3.** ANOVA for the regression equation of Lignin production of NaOH pretreated corn husk biomass

| Source | Sum of Squares | df | Mean Square | F-value | p-value |  |
| --- | --- | --- | --- | --- | --- | --- |
| Model | 207.5 | 14 | 14.8 | 6.94 | 0.0002 | significant |
| A-SC | 49.3 | 1 | 49.3 | 23.1 | 0.0002 |  |
| B-ALK | 2.38 | 1 | 2.38 | 1.11 | 0.307 |  |
| C-Temp | 18.1 | 1 | 18.1 | 8.48 | 0.010 |  |
| D-IT | 4.47 | 1 | 4.47 | 2.09 | 0.167 |  |
| AB | 18.86 | 1 | 18.9 | 8.82 | 0.009 |  |
| AC | 37.6 | 1 | 37.6 | 17.59 | 0.0007 |  |
| AD | 7.24 | 1 | 7.24 | 3.39 | 0.084 |  |
| BC | 8.12 | 1 | 8.12 | 3.80 | 0.068 |  |
| BD | 0.673 | 1 | 0.673 | 0.315 | 0.582 |  |
| CD | 0.105 | 1 | 0.105 | 0.049 | 0.826 |  |
| A² | 42.7 | 1 | 42.7 | 19.9 | 0.0004 |  |
| B² | 23.8 | 1 | 23.8 | 11.1 | 0.004 |  |
| C² | 35.7 | 1 | 35.7 | 16.7 | 0.0009 |  |
| D² | 29.3 | 1 | 29.3 | 13.7 | 0.002 |  |
| Residual | 34.2 | 16 | 2.14 |  |  |  |
| Lack of Fit | 9.18 | 8 | 1.15 | 0.367 | 0.911 | not significant |
| Pure Error | 25.0 | 8 | 3.13 |  |  |  |
| Cor Total | 241.7 | 30 |  |  |  |  |

| **Std. Dev.** | **Mean** | **C.V. %** | **R²** | **Adjusted R²** | **Predicted R²** | **Adeq Precision** |
| --- | --- | --- | --- | --- | --- | --- |
| 1.46 | 12.6 | 11.6 | 0.858 | 0.734 | 0.508 | 10.2 |

**Table S4.** ANOVA for the regression equation of cellulose production of H_2_SO_4_ pretreated corn husk biomass

| Source | | Sum of Squares | | df | | Mean Square | | F-value | | p-value |  | |  |
| --- | --- | --- | --- | --- | --- | --- | --- | --- | --- | --- | --- | --- | --- |
| Model | | 1.517+05 | | 14 | | 10838.9 | | 3.21 | | 0.018 | significant | |  |
| A-SC | | 23787.7 | | 1 | | 23787.7 | | 7.05 | | 0.018 |  | |  |
| B-Acidic conc | | 23918.5 | | 1 | | 23918.5 | | 7.09 | | 0.018 |  | |  |
| C-IT | | 40264.8 | | 1 | | 40264.8 | | 11.9 | | 0.004 |  | |  |
| D-T | | 299.4 | | 1 | | 299.4 | | 0.088 | | 0.770 |  | |  |
| AB | | 26303.7 | | 1 | | 26303.7 | | 7.79 | | 0.014 |  | |  |
| AC | | 9854.9 | | 1 | | 9854.9 | | 2.92 | | 0.109 |  | |  |
| AD | | 7863.8 | | 1 | | 7863.8 | | 2.33 | | 0.149 |  | |  |
| BC | | 3916.2 | | 1 | | 3916.2 | | 1.16 | | 0.299 |  | |  |
| BD | | 1024.3 | | 1 | | 1024.3 | | 0.303 | | 0.590 |  | |  |
| CD | | 2105.0 | | 1 | | 2105.0 | | 0.623 | | 0.442 |  | |  |
| A² | | 2817.3 | | 1 | | 2817.3 | | 0.834 | | 0.376 |  | |  |
| B² | | 15950.5 | | 1 | | 15950.5 | | 4.73 | | 0.047 |  | |  |
| C² | | 2066.3 | | 1 | | 2066.3 | | 0.612 | | 0.447 |  | |  |
| D² | | 29547.9 | | 1 | | 29547.9 | | 8.75 | | 0.010 |  | |  |
| Residual | | 47249.8 | | 14 | | 3374.9 | |  | |  |  | |  |
| Lack of Fit | | 34337.6 | | 11 | | 3121.6 | | 0.725 | | 0.699 | not significant | |  |
| Pure Error | | 12912.2 | | 3 | | 4304.0 | |  | |  |  | |  |
| Cor Total | | 1.99+05 | | 28 | |  | |  | |  |  | |  |
| **Std. Dev.** | **Mean** | | **C.V. %** | | **R²** | | **Adjusted R²** | | **Predicted R²** | | | **Adeq Precision** | |
| 58.09 | 438.6 | | 13.24 | | 0.762 | | 0.525 | | 0.023 | | | 6.939 | |

**Table S5.** ANOVA for the regression equation of hemicellulose production of H_2_SO_4_ pretreated corn husk biomass

| **Source** | **Sum of Squares** | **df** | **Mean Square** | **F-value** | **p-value** |  |
| --- | --- | --- | --- | --- | --- | --- |
| **Model** | 3848.4 | 14 | 274.9 | 3.04 | 0.023 | significant |
| A-SC | 1164.6 | 1 | 1164.6 | 12.9 | 0.003 |  |
| B-Acidic conc | 765.3 | 1 | 765.3 | 8.46 | 0.011 |  |
| C-IT | 1040.4 | 1 | 1040.4 | 11.5 | 0.0044 |  |
| D-T | 1273.9 | 1 | 1273.9 | 14.1 | 0.002 |  |
| AB | 162.3 | 1 | 162.3 | 1.79 | 0.201 |  |
| AC | 1124.1 | 1 | 1124.1 | 12.4 | 0.003 |  |
| AD | 1976.5 | 1 | 1976.5 | 21.8 | 0.0004 |  |
| BC | 170.5 | 1 | 170.5 | 1.9 | 0.191 |  |
| BD | 19.7 | 1 | 19.7 | 0.218 | 0.647 |  |
| CD | 316.5 | 1 | 316.5 | 3.50 | 0.082 |  |
| A² | 630.2 | 1 | 630.2 | 6.97 | 0.019 |  |
| B² | 64.9 | 1 | 64.9 | 0.718 | 0.411 |  |
| C² | 924.6 | 1 | 924.6 | 10.2 | 0.006 |  |
| D² | 1194.3 | 1 | 1194.3 | 13.2 | 0.003 |  |
| **Residual** | 1265.8 | 14 | 90.4 |  |  |  |
| Lack of Fit | 1045.2 | 11 | 95.0 | 1.29 | 0.467 | not significant |
| Pure Error | 220.5 | 3 | 73.5 |  |  |  |
| **Cor Total** | 5114.3 | 28 |  |  |  |  |

| **Std. Dev.** | **Mean** | **C.V. %** | **R²** | **Adjusted R²** | **Predicted R²** | **Adeq Precision** |
| --- | --- | --- | --- | --- | --- | --- |
| 9.51 | 117.8 | 8.07 | 0.752 | 0.505 | 0.374 | 7.48 |

**Table S6.** ANOVA for the regression equation of Lignin production of H_2_SO_4_ pretreated corn husk biomass

| **Source** | **Sum of Squares** | **df** | **Mean Square** | **F-value** | **p-value** |  |
| --- | --- | --- | --- | --- | --- | --- |
| **Model** | 48.5 | 14 | 3.46 | 3.21 | 0.018 | significant |
| A-SC | 4.35 | 1 | 4.35 | 4.04 | 0.064 |  |
| B-Acidic conc | 4.38 | 1 | 4.38 | 4.07 | 0.063 |  |
| C-IT | 3.84 | 1 | 3.84 | 3.56 | 0.08 |  |
| D-T | 2.33 | 1 | 2.33 | 2.17 | 0.163 |  |
| AB | 16.6 | 1 | 16.6 | 15.4 | 0.002 |  |
| AC | 0.053 | 1 | 0.053 | 0.048 | 0.828 |  |
| AD | 8.93 | 1 | 8.93 | 8.29 | 0.012 |  |
| BC | 3.40 | 1 | 3.40 | 3.16 | 0.097 |  |
| BD | 0.562 | 1 | 0.562 | 0.522 | 0.481 |  |
| CD | 0.075 | 1 | 0.075 | 0.07 | 0.795 |  |
| A² | 1.38 | 1 | 1.38 | 1.28 | 0.277 |  |
| B² | 10.1 | 1 | 10.1 | 9.34 | 0.009 |  |
| C² | 5.18 | 1 | 5.18 | 4.81 | 0.045 |  |
| D² | 4.16 | 1 | 4.16 | 3.86 | 0.07 |  |
| **Residual** | 15.1 | 14 | 1.1 |  |  |  |
| Lack of Fit | 13.4 | 11 | 1.2 | 2.20 | 0.28 | not significant |
| Pure Error | 1.66 | 3 | 0.553 |  |  |  |
| **Cor Total** | 63.6 | 28 |  |  |  |  |

| **Std. Dev.** | **Mean** | **C.V. %** | **R²** | **Adjusted R²** | **Predicted R²** | **Adeq Precision** |
| --- | --- | --- | --- | --- | --- | --- |
| 1.04 | 14.7 | 7.06 | 0.763 | 0.525 | 0.283 | 6.242 |

**Table S7:** Pretreatment Efficiency of Corn Husk Biomass

| **Pretreatment** | **Lignin Removal (%)** | **Cellulose Recovery (%)** | **Hemicellulose Recovery (%)** |
| --- | --- | --- | --- |
| Alkali (NaOH) | 35 | 48 | 39 |
| Acid (H_2_SO_4_) | 30 | 45 | 34 |
| Ultrasonication + Alkali (NaOH) | 49 | 51 | 46 |

**Table S8.** ANOVA for the regression equation of glucose production of enzymatic hydrolysed cornhusk biomass

| **Source** | | **Sum of Squares** | | **df** | | **Mean Square** | | **F-value** | | **p-value** | |  | |
| --- | --- | --- | --- | --- | --- | --- | --- | --- | --- | --- | --- | --- | --- |
| **Model** | | 53115.3 | | 14 | | 3793.9 | | 10.4 | | 0.0001 | | significant | |
| A-pH | | 2776.6 | | 1 | | 2776.6 | | 7.64 | | 0.017 | |  | |
| B-Temp | | 16621.4 | | 1 | | 16621.4 | | 45.8 | | < 0.0001 | |  | |
| C-EC | | 1246.7 | | 1 | | 1246.7 | | 3.43 | | 0.088 | |  | |
| D-IT | | 4547.9 | | 1 | | 4547.9 | | 12.5 | | 0.004 | |  | |
| AB | | 12432.3 | | 1 | | 12432.3 | | 34.2 | | < 0.0001 | |  | |
| AC | | 9971.5 | | 1 | | 9971.5 | | 27.4 | | 0.0002 | |  | |
| AD | | 289.0 | | 1 | | 289.0 | | 0.795 | | 0.389 | |  | |
| BC | | 6090.4 | | 1 | | 6090.4 | | 16.7 | | 0.001 | |  | |
| BD | | 1332.2 | | 1 | | 1332.2 | | 3.67 | | 0.079 | |  | |
| CD | | 938.9 | | 1 | | 938.9 | | 2.6 | | 0.133 | |  | |
| A² | | 6195.6 | | 1 | | 6195.6 | | 17.1 | | 0.001 | |  | |
| B² | | 348.5 | | 1 | | 348.5 | | 0.959 | | 0.346 | |  | |
| C² | | 307.4 | | 1 | | 307.4 | | 0.846 | | 0.375 | |  | |
| D² | | 11823.2 | | 1 | | 11823.2 | | 32.55 | | < 0.0001 | |  | |
| **Residual** | | 4358.5 | | 12 | | 363.21 | |  | |  | |  | |
| Lack of Fit | | 2785.9 | | 10 | | 278.6 | | 0.354 | | 0.893 | | not significant | |
| Pure Error | | 1572.7 | | 2 | | 786.3 | |  | |  | |  | |
| **Cor Total** | | 57473.8 | | 26 | |  | |  | |  | |  | |
| **Std. Dev.** | **Mean** | | **C.V. %** | | **R²** | | **Adjusted R²** | | **Predicted R²** | | **Adeq Precision** | |  |
| 19.06 | 300.9 | | 6.33 | | 0.924 | | 0.836 | | 0.565 | | 12.679 | |  |

**Table S9.** ANOVA for the regression equation of xylose production of enzymatic hydrolysed cornhusk biomass

| **Source** | | **Sum of Squares** | | **df** | | **Mean Square** | | **F-value** | | **p-value** | |  |  |
| --- | --- | --- | --- | --- | --- | --- | --- | --- | --- | --- | --- | --- | --- |
| **Model** | | 1162.6 | | 14 | | 83.0 | | 7.36 | | 0.0003 | | significant |  |
| A-Enzyme conc. | | 60.2 | | 1 | | 60.2 | | 5.33 | | 0.037 | |  |  |
| B-IT | | 0.399 | | 1 | | 0.399 | | 0.035 | | 0.853 | |  |  |
| C-pH | | 10.5 | | 1 | | 10.5 | | 0.9349 | | 0.350 | |  |  |
| D-Temp. | | 44.5 | | 1 | | 44.5 | | 3.95 | | 0.067 | |  |  |
| AB | | 0.632 | | 1 | | 0.632 | | 0.056 | | 0.816 | |  |  |
| AC | | 113.9 | | 1 | | 113.9 | | 10.1 | | 0.007 | |  |  |
| AD | | 379.9 | | 1 | | 379.9 | | 33.7 | | < 0.0001 | |  |  |
| BC | | 2.71 | | 1 | | 2.71 | | 0.24 | | 0.632 | |  |  |
| BD | | 145.6 | | 1 | | 145.6 | | 12.9 | | 0.003 | |  |  |
| CD | | 28.4 | | 1 | | 28.4 | | 2.52 | | 0.135 | |  |  |
| A² | | 6.25 | | 1 | | 6.25 | | 0.554 | | 0.469 | |  |  |
| B² | | 63.5 | | 1 | | 63.5 | | 5.63 | | 0.033 | |  |  |
| C² | | 153.1 | | 1 | | 153.1 | | 13.6 | | 0.003 | |  |  |
| D² | | 207.5 | | 1 | | 207.5 | | 18.4 | | 0.0007 | |  |  |
| **Residual** | | 157.9 | | 14 | | 11.3 | |  | |  | |  |  |
| Lack of Fit | | 55.7 | | 10 | | 5.57 | | 0.217 | | 0.977 | | not significant |  |
| Pure Error | | 102.7 | | 4 | | 25.6 | |  | |  | |  |  |
| **Cor Total** | | 1320.6 | | 28 | |  | |  | |  | |  |  |
| **Std. Dev.** | **Mean** | | **C.V. %** | | **R²** | | **Adjusted R²** | | **Predicted R²** | | **Adeq Precision** | | |
| 3.36 | 28.4 | | 11.8 | | 0.880 | | 0.760 | | 0.636 | | 9.92 | | |

**Table S10.** A comparative study of bioethanol production from different agrowastes using various pretreatment, enzymatic hydrolysis and fermentation techniques

| **Agrowastes** | **Pretreatment method and Severity** | **Enzyme loading (FPU/g-dw)** | **TRS** | **Fermentative Microorganisms** | **Mode of fermentation** | **Ethanol production (g/L)** | **References** |
| --- | --- | --- | --- | --- | --- | --- | --- |
| Corn cobs | 70% H₂SO₄ acid pretreatment  **Very high** (concentrated acid) | NA  (β‑glucanase + β‑glucosidase) | 11.33 mg/mL | -- | Batch fermentation | 52.3 | Sokan-Adeaga et al., 2024 |
| Corn stover | Supercritical fluid extraction + steam explosion  **High** (thermal–pressure assisted) | NA | NA | 1. *cerevisiae* and *P.stipitis* | Batch co‑fermentation (C5/C6 sugars) | 15.36 | Chen et al., 2017 |
| Corn stover | Microwave‑assisted ionic liquid [DIP][HSO₄]  **High**(ionic liquid + microwave) | ~15 FPU/g-dw (Cellic CTec2) | 80.88 g L−1 | C5/C6 co-assimilation *Saccharomycescerevisiae* YL23 | Batch fermentation with C5/C6 co‑assimilation | 34.35 | Wang et al., 2024 |
| Sweet sorghum residue | Alkali pretreatment (NaOH)  **Moderate** | ~20 FPU/g-dw (cellulase + xylanase) | 42.7 mg/g | *S.cerevisiae* and *P.stipitis* | Batch co‑fermentation | 16.8 | Punia and Kumar, 2025 |
| Agricultural wastes | 2% NaOH + H₂O₂ (9:1) followed by organosolv  **Moderate high** | ~25 FPU/g-dw (cellulase:xylanase = 3:2) | 22.53 mg/g | *S.cerevisiae* and *P.stipitis* | Batch co‑fermentation | 28.44 | Sharma and Sharma 2024 |
| Cotton stalk | High‑pressure alkaline (NaOH + H₂O₂)  **High** | 30 FPU/g-dw | 241 mg/g | *S.cerevisiae* | Batch fermentation | 3.96 | Malik et al., 2020 |
| Corn husk | Microwave‑assisted 0.5 M NaOH | ~20 FPU/g-dw (*Fusarium* cellulase) |  | *Fusarium oxysporum* and *S.cerevisiae* | Sequential hydrolysis and fermentation (SHF) | 50.3 | Sharma et al., 2018 |
| Corn husk | Ultrasonication combined alkali pretreatment  **Moderate–high** | ~25–30 FPU/g-dw (Celluclast + Viscozyme) | 89.2 g/L | *S.cerevisiae* and *P.pastories* | Simultaneous saccharification and fermentation (SSF) | 37.8 | Present study |
